# CRISPR-Cas9 screen of E3 ubiquitin ligases identifies TRAF2 and UHRF1 as regulators of HIV latency in primary human T cells

**DOI:** 10.1128/mbio.02222-23

**Published:** 2024-02-27

**Authors:** Ujjwal Rathore, Paige Haas, Vigneshwari Easwar Kumar, Joseph Hiatt, Kelsey M. Haas, Mehdi Bouhaddou, Danielle L. Swaney, Erica Stevenson, Lorena Zuliani-Alvarez, Michael J. McGregor, Autumn Turner-Groth, Charles Ochieng' Olwal, Yaw Bediako, Hannes Braberg, Margaret Soucheray, Melanie Ott, Manon Eckhardt, Judd F. Hultquist, Alexander Marson, Robyn M. Kaake, Nevan J. Krogan

**Affiliations:** 1 Gladstone Institutes, San Francisco, California, USA; 2 Department of Microbiology and Immunology, University of California, San Francisco, California, USA; 3 Innovative Genomics Institute, University of California, Berkeley, California, USA; 4 Quantitative Biosciences Institute (QBI), University of California, San Francisco, California, USA; 5 Department of Cellular and Molecular Pharmacology, University of California, San Francisco, California, USA; 6 Medical Scientist Training Program, University of California, San Francisco, California, USA; 7 Biomedical Sciences Graduate Program, University of California, San Francisco, California, USA; 8 West African Centre for Cell Biology of Infectious Pathogens (WACCBIP), College of Basic and Applied Sciences, University of Ghana, Accra, Ghana; 9 Department of Biochemistry, Cell & Molecular Biology, College of Basic & Applied Sciences, University of Ghana, Accra, Ghana; 10 Division of Infectious Diseases, Northwestern University Feinberg School of Medicine, Chicago, Illinois, USA; 11 Center for Pathogen Genomics and Microbial Evolution, Institute for Global Health, Northwestern University Feinberg School of Medicine, Chicago, Illinois, USA; 12 Department of Medicine, University of California, San Francisco, California, USA; 13 Diabetes Center, University of California, San Francisco, California, USA; 14 UCSF Helen Diller Family Comprehensive Cancer Center, University of California, San Francisco, California, USA; 15 Parker Institute for Cancer Immunotherapy, University of California, San Francisco, California, USA; 16 Institute for Human Genetics, University of California, San Francisco, California, USA; University of Pittsburgh School of Medicine, Pittsburgh, Pennsylvania, USA; Stanford University School of Medicine, Stanford, California, USA

**Keywords:** human immunodeficiency virus, ubiquitin E3 ligases, HIV latency, CRISPR screen, resting primary T cells

## Abstract

During HIV infection of CD4+ T cells, ubiquitin pathways are essential to viral replication and host innate immune response; however, the role of specific E3 ubiquitin ligases is not well understood. Proteomics analyses identified 116 single-subunit E3 ubiquitin ligases expressed in activated primary human CD4+ T cells. Using a CRISPR-based arrayed spreading infectivity assay, we systematically knocked out 116 E3s from activated primary CD4+ T cells and infected them with NL4-3 GFP reporter HIV-1. We found 10 E3s significantly positively or negatively affected HIV infection in activated primary CD4+ T cells, including UHRF1 (pro-viral) and TRAF2 (anti-viral). Furthermore, deletion of either TRAF2 or UHRF1 in three JLat models of latency spontaneously increased HIV transcription. To verify this effect, we developed a CRISPR-compatible resting primary human CD4+ T cell model of latency. Using this system, we found that deletion of TRAF2 or UHRF1 initiated latency reactivation and increased virus production from primary human resting CD4+ T cells, suggesting these two E3s represent promising targets for future HIV latency reversal strategies.

**IMPORTANCE:**

HIV, the virus that causes AIDS, heavily relies on the machinery of human cells to infect and replicate. Our study focuses on the host cell’s ubiquitination system which is crucial for numerous cellular processes. Many pathogens, including HIV, exploit this system to enhance their own replication and survival. E3 proteins are part of the ubiquitination pathway that are useful drug targets for host-directed therapies. We interrogated the 116 E3s found in human immune cells known as CD4+ T cells, since these are the target cells infected by HIV. Using CRISPR, a gene-editing tool, we individually removed each of these enzymes and observed the impact on HIV infection in human CD4+ T cells isolated from healthy donors. We discovered that 10 of the E3 enzymes had a significant effect on HIV infection. Two of them, TRAF2 and UHRF1, modulated HIV activity within the cells and triggered an increased release of HIV from previously dormant or “latent” cells in a new primary T cell assay. This finding could guide strategies to perturb hidden HIV reservoirs, a major hurdle to curing HIV. Our study offers insights into HIV-host interactions, identifies new factors that influence HIV infection in immune cells, and introduces a novel methodology for studying HIV infection and latency in human immune cells.

## INTRODUCTION

Human immunodeficiency virus is dependent on a complex network of host cell machinery to establish infection, replicate, and transmit in humans. HIV primarily infects CD4+ T cells via binding to the CD4 receptor and either CCR5 or CXCR4 co-receptors. Following cellular entry and uncoating, the HIV RNA genome is reverse transcribed into DNA which integrates into the host genome and is transcribed into viral mRNA. Viral mRNA is translated into viral proteins, which act in concert with host proteins to package viral RNA and assemble HIV virions that are released via budding (1, 2). Untreated HIV leads to the development of acquired immunodeficiency syndrome, which is marked by CD4+ T cell depletion and impaired immune responses to subsequent infections. Currently, antiretroviral therapy (ART) consists of a combination of multiple drugs that inhibit key parts of the HIV life cycle and suppress viral load, preventing the progression to AIDS and decreasing the risk of transmission to others. However, ART is not an HIV cure as it does not eliminate the reservoir of infected cells that are not producing virions. These latently infected cells are not killed by viral toxicity or immune clearance and can persist for decades, with the ability to randomly reactivate when treatment is stopped (3, 4). For this reason, ART must be lifelong. An improved understanding of how HIV modulates host cell responses is critical to identifying new therapeutic targets that can lead to a cure.

One of the important processes involved in HIV-host interactions is the cell ubiquitination machinery. The host ubiquitination signaling pathway is essential for a number of cellular processes including protein degradation and quality control, cellular localization, and molecular interactions, and therefore, many pathogens hijack this system to promote their own replication and survival (5, 6). Ubiquitin as a signaling molecule is covalently conjugated to protein substrates in an ATP-dependent process catalyzed by three enzymes: E1 ubiquitin-activating enzymes, E2 ubiquitin-conjugating enzymes, and E3 ubiquitin ligases which catalyze the transfer of ubiquitin to the protein substrate. The mechanism by which E3 ligases attach ubiquitin to the protein substrate is determined by their type: a really interesting new gene (RING) facilitates ubiquitin transfer directly from the E2 to the substrate, human thyroid receptor-interacting protein (HECT) binds ubiquitin themselves before transfer to the substrate, and ring-in-between-ring (RBR) contains RING domains but bind ubiquitin themselves similar to HECT E3s (7).

The importance of ubiquitin signaling during HIV infection has been reflected in genetic screens of host proteins where the genes identified are enriched for ubiquitination machinery and ubiquitin-regulated pathways (8–10). In addition, proteomic studies of HIV-host protein-protein interactions have found host interactors that are enriched for roles in ubiquitin signaling (11). E3 ubiquitin ligases have been shown to support and block HIV infection during the viral replication cycle. For example, after entry, the HIV protein Vpu targets the host receptor CD4 for degradation via βTRCP, a subunit of a multisubunit E3 ubiquitin ligase (12). The cullin 5 RING E3 ligase (CRL5) complex is hijacked by the HIV protein Vif to promote the degradation of host APOBEC3 (A3) restriction factors (13, 14). Unchecked, this family of cytosine deaminases binds to and hypermutates the HIV genome, effectively producing non-infectious virions (15). An additional E3 ligase, ARIH2, helps catalyze the mono-ubiquitylation of A3 proteins prior to subsequent poly-ubiquitylation and proteasomal degradation (16). Ubiquitin pathways also regulate a number of key processes important for HIV latency reversal, including epigenetic regulation and transcription (17, 18).

E3 ubiquitin ligases represent a good therapeutic target for HIV as they are more selective than E1s and E2s (19), making them excellent therapeutics with limited off-target effects. Given their importance in HIV replication and host processes related to HIV pathogenesis, there is a need for a comprehensive study that clearly identifies E3s important to HIV infection in physiologically relevant primary CD4+ T cells, as most studies have been performed in immortalized cell lines. The knowledge gained from such a study can not only inform about novel HIV-host interactions but might also provide new targets for disrupting the latent HIV reservoir, the main block to HIV cure strategies. While many latency reversal agents (LRAs) have been identified (20), they usually reactivate less than 5% of latently infected CD4+ T cells (21). Without a universally penetrant combination therapy strategy, there is an urgent need for the discovery of novel complementary and highly potent LRAs (22). Toward this end, elucidating host genes, proteins, and pathways that govern latency maintenance and reversal is essential for identifying synergistic combinatorial LRA therapies.

Here, we study the functional role of E3 ubiquitin ligases in the context of HIV-1 infection in physiologically relevant primary CD4+ T cells isolated from healthy human donors. In order to determine E3 ligases important for regulating HIV infection, we first identified which E3 proteins are expressed in primary CD4+ T cells. Mass spectrometry analysis identified 116 single-subunit E3s, which we systematically deleted in an arrayed format in primary CD4+ T cells and challenged with HIV infection. Through this assay, we identified 10 E3s that regulate HIV infection both positively and negatively, and using network propagation, we mapped host pathways connecting HIV-relevant genes, proteins, and pathways to our functional E3s. Our E3-HIV network identified enrichments in TNF and non-canonical NF-κB pathways, which have been involved in HIV transcription and latency. Excitingly, testing these E3s in three Jurkat (JLat) cell line models of latency revealed that TRAF2 knockout alone reverses HIV latency, as does the knockout of the epigenetic regulator UHRF1. Due to the limitations of the JLat latency model, we developed a novel, CRISPR-compatible, and physiologically relevant primary human resting CD4+ T cell model of latency. Consistent with our results in JLat models, we found that TRAF2 and UHRF1 knockouts increased HIV transcription intracellularly. Unlike cell-line models of latency, our resting T cell model of latency makes use of a fully replication-competent HIV strain allowing us to discover that TRAF2 and UHRF1 knockouts also result in an increase in HIV production and release from resting primary CD4+ T cells isolated from six healthy human donors. Thus, we have identified two E3 ligases: TRAF2 and UHRF1 as exciting candidate drug targets for the development of novel latency-reversing agents for an HIV cure.

## RESULTS

### Liquid chromatography tandem mass spectrometry analysis identifies 116 E3 ligases expressed in activated primary human CD4+ T cells

**Fig 1:**
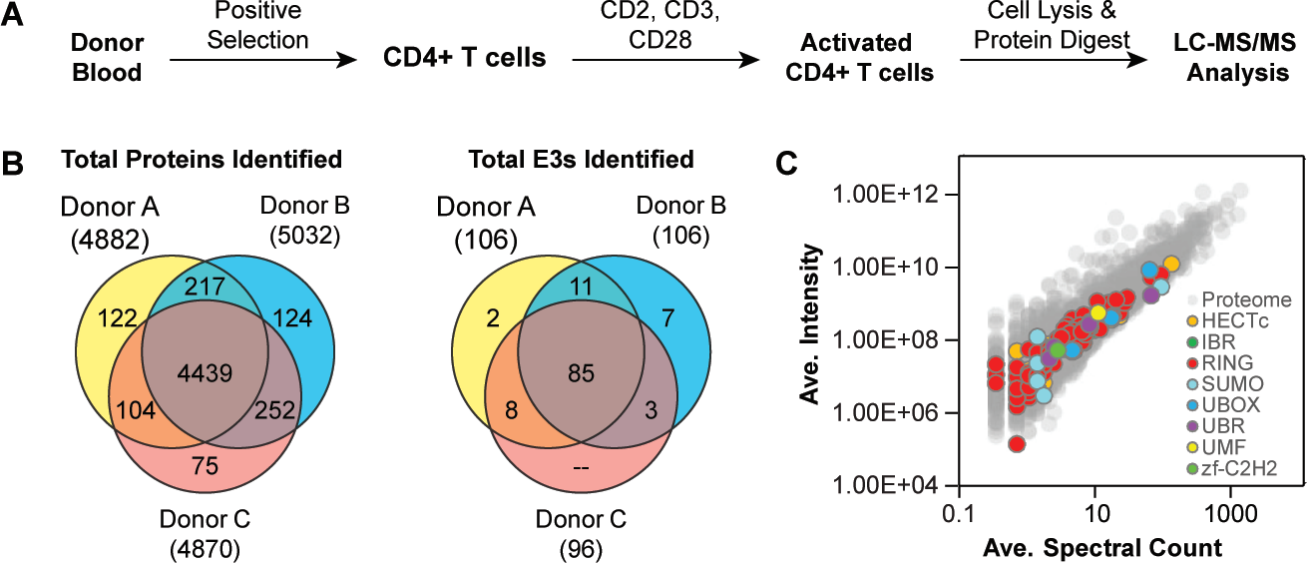
LC-MS/MS analysis identifies 116 E3 ligases expressed in activated primary human CD4+ T cells. (A) Workflow describing the proteomic identification of expressed E3s in activated primary CD4+ T cells from three healthy human donors. (B) Venn diagrams showing the overlap of the 5,333 total proteins (left) and 116 E3s (right) expressed in primary activated CD4+ T cells from three healthy human donors. (**C**) Scatter plot showing protein abundance by average (ave.) spectral count and average intensity for each protein identified in all three donors (for proteins not identified in all three donors, the missing value was ignored in the average calculation). E3 ligases are represented by colored markers according to their type (see legend inset), and non-E3 ligase proteins are represented by gray markers.

In order to characterize the E3 ligases (E3s) relevant to HIV infection, we first identified which E3 proteins are expressed in the major cell type infected by HIV: activated primary human CD4+ T cells. To this end, CD4+ T cells were isolated by positive selection from blood donated by three healthy individuals, activated with anti-CD2, anti-CD3, and anti-CD28 antibodies, and harvested after 6 days. Activated CD4+ T cells were lysed, proteins were proteolytically digested, and resulting peptides were de-salted and analyzed by mass spectrometry (liquid chromatography tandem mass spectrometry [LC-MS/MS] analysis) (Fig. 1A). To be as inclusive as possible for our initial screen, we considered as expressed in CD4+ T cells any protein that was identified in any of the three donors by at least one unique peptide. In total, we identified 5,333 unique proteins across all three donors, with 5,012 shared in at least two donors, and 4,439 identified in all three (Fig. 1B; Table S1).

For this study, we were primarily interested in E3 ligases that function as single subunits rather than multisubunit ligases (e.g., cullin-ring ligases) that would presumably be more challenging to deconvolute the phenotypes obtained after gene deletion. Though a number of sources exist characterizing E3 ligases (23–25), we cross-referenced our list of 5,333 expressed proteins against a database of 377 annotated human E3 ligases since it provided a unique reference of E3 classification that could help prioritize E3s that function as single subunits (25). In total, we identified 116 single-subunit E3 ligases that were expressed at the protein level in primary activated CD4+ T cells from three healthy human donors. A similar number of E3 ligases were detected in each donor, with 106 detected in Donor A, 106 in Donor B, and 96 in Donor C. There was considerable donor-to-donor overlap, with 85 E3s identified in all three donors and 22 E3s identified in two donors, while 9 E3s were identified in only one donor (Fig. 1B). We detected E3s measured across low- and-high abundance ranges relative to all proteins identified in the data set, with the majority of E3s falling at mid-to-low abundance (Fig. 1C). These proteins reflect different E3 types and span a variety of different mechanisms of ubiquitin ligation. We detected all but two types that are classified in the original E3 database: RING (including IBR), HECT, UBR, UBox, and zf-C2H2 E3s (Table S1). We detected 86 RING E3s (including 2 IBR E3s) out of 331 in the database, 12 out of 30 HECT E3s, 4 out of 5 UBR E3s, 4 out of 5 UBox E3s, and 1 out of 2 zf-C2H2 E3s (Fig. 1C). In addition to this list cross-checked against the 377 annotated human E3s, we also investigated E3s that ligate ubiquitin-like molecules including small ubiquitin-like modifier (SUMO) family proteins and ubiquitin-fold modifier 1 (UMF1). Literature-curated and database-guided characterization identified an additional nine E3 ligases expressed in our CD4+ T cell proteomics data set: 8 SUMO E3s and 1 UMF E3 (Fig. 1C).

### CRISPR-Cas9 gene knockouts in activated primary human CD4+ T cells identify novel E3s that regulate HIV infection

**Fig 2:**
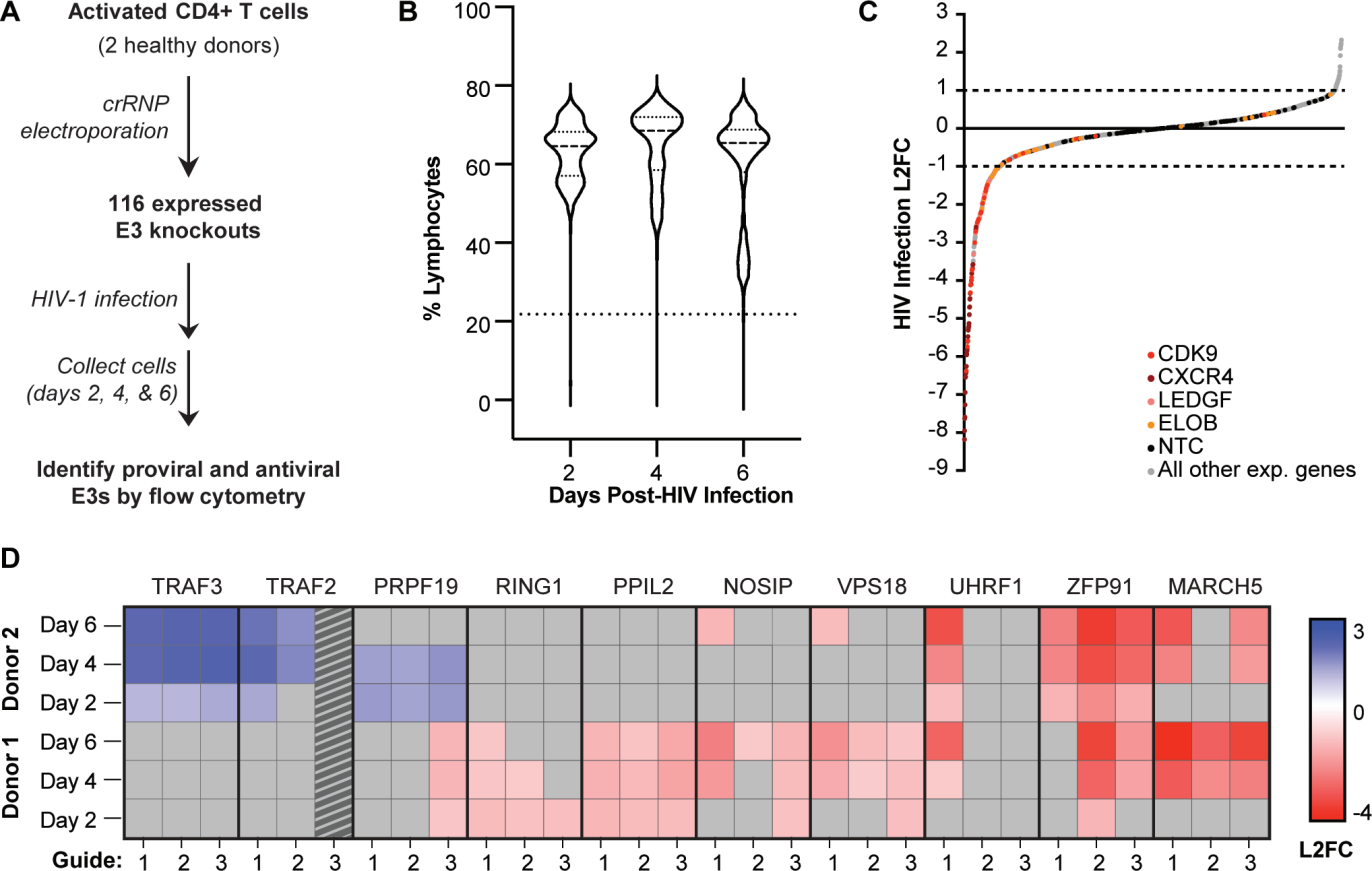
CRISPR-Cas9 knockouts in primary human CD4+ T cells identify novel E3s that regulate HIV infection. (A) Workflow showing CRISPR knockout and screening of 116 E3 knockouts for a functional role in HIV-1 infection in primary activated CD4+ T cells from healthy human donors. (**B**) Flow cytometry quantification of the percentage of lymphocytes across all donors, guides, and technical replicates of the E3 knockouts on days 2, 4, and 6 post-HIV infection. (**C**) Flow cytometry quantification of HIV infection (% GFP-positive cells normalized to NTCs and log2 transformed) for all gene knockouts, in all donors, at 2, 4, and 6 days after HIV-1 infection. Known dependency factor controls are marked in color, NTCs are marked in black, and all E3s are colored gray. (**D**) Heatmap showing HIV infection for 10 E3 hits. Hits were defined as a gene knockout that yielded an HIV infection log2 fold change (L2FC) compared with NTC of ≥1 or ≤−1 in the same direction in at least two time points for two guides within the same donor or the same guide in two donors. Data points with a L2FC < 1 or >−1 are colored gray. Data from TRAF2 guide 3 were removed as it fell below our viability estimate filter.

In order to identify which E3s act in a pro-viral or anti-viral capacity against HIV-1 in primary activated CD4+ T cells, we used an arrayed CRISPR-knockout (KO)-compatible spreading HIV-1 infection assay (26, 27). Briefly, we isolated and activated CD4+ T cells from healthy human donors, electroporated pre-formed CRISPR-ribonucleoproteins (crRNPs) in a 96-well arrayed format in three technical replicates, and infected them with replication-competent HIV-1 NL4-3 Nef:IRES:GFP (Fig. 2A) (28). Every plate consisted of an outer-well border plated with media (to limit edge effects) and included three control wells with individual non-targeting control (NTC) guides selected to not align to any protospacer region of the human genome and which thus should not edit the genome or affect HIV infection. Each plate also included four positive controls, which are known dependency factors for HIV-1 NL4-3: CXCR4 (29–31), CDK9 (32, 33), LEDGF (34), and ELOB (35). The remaining wells targeted E3 ligases, with each E3 gene being targeted by three individually designed guide RNAs (gRNAs) to increase knockout efficiency (Table S2). Samples were collected at 2, 4, and 6 days post-infection and infection was measured by flow cytometry counting the percentage of cells that were GFP positive (Fig. 2A).

In order to avoid confounding interpretations due to cell toxicity or death associated with gene KOs, cell viability was estimated by counting the percentage of live lymphocytes. Most guides displayed limited toxicity (Fig. 2B), and the samples in the bottom 1% of the data set for both percentage and counts of live lymphocytes were removed from analysis due to their lower viability (Fig. S1). In total, 140 individual replicates were removed from the data set across guides and donors for individual wells, due to low viability (Table S3; Fig. S1). The median percentage of HIV infection varied between donors and replicate plates; therefore, to standardize across experiments, we normalized by the donor and plate. To this end, the percentage of GFP-positive cells was measured for each well of every plate, with each plate always containing 3 individual wells having 1 unique NTC each, 4 positive controls targeting HIV-1 dependency factors, and 65 E3 targeting guides. Of the 116 expressed E3s, TRIM21 and PHRF1 did not have a pre-designed CRISPR guide RNA available for order and were therefore removed from our study. However, we included two additional E3s not detected by proteomic analysis: TRAF3 and BIRC3. These two proteins function as components of the NF-κB-inducing kinase (NIK) regulatory complex for which we had identified other members (e.g., TRAF2 and BIRC2) in our proteomic analysis and wanted to probe this complex further. For every well, the percentage of GFP-positive cells was first normalized to the median result of three NTCs. The normalized values were then averaged across the three technical replicate plates and log2 transformed.

All controls worked as expected, NTCs had a minimal effect on HIV infection (less than a log2 fold change [L2FC] of 1), whereas knocking out positive control HIV dependency factors such as CXCR4. LEDGF, CDK9, and ELOB decreased HIV infection by at least twofold (Fig. 2C). Based on these results from the NTCs and positive controls, we set a threshold of (L2FC > 1 or <−1) for calling an E3 a hit as described previously (27). We considered an E3 a hit if the knockout passed our L2FC > 1 or <−1 threshold in the same direction at two time points in at least two guides in the same donor or the same guide in two donors. By defining biological replicates in this primary screen as either the same guide in two donors or two guides within the same donor, we maximized the likelihood of observing hits which can be validated for biological significance in complementary follow-up experiments. This less-stringent threshold accounts for the inherent variability in performing large screens in primary cells isolated from multiple donors. For example, diverse susceptibility to HIV infection under identical conditions across donors could result in HIV infection saturation in a specific donor, making it difficult to observe knockouts that increase HIV infection in that donor. In this case, having biologically distinct replicates from multiple guides targeting the same gene in at least one donor provides greater confidence in borderline hits. Donor variability in the form of single nucleotide polymorphisms on the other hand might result in differences in gene editing efficiency per guide. For example, if only one of three guides targeting a gene achieves highly efficient editing in a specific donor, the gene’s effect on HIV infection might be unclear. In this case, having two donors as biological replicates for the same guide provides higher confidence in the putative hit. Using these criteria, we found 10 E3s that affect HIV infection: seven with pro-viral activity (MARCH5, ZFP91, UHRF1, VPS18, NOSIP, PPIL2, and RING1) and three with anti-viral activity (TRAF2, TRAF3, and PRPF19) (Fig. 2D) in activated primary CD4+ T cells. Notably, 5 of these E3s make a more stringent cutoff of a L2FC > 1.5 or <−1.5 and represent higher-confidence hits: TRAF2, TRAF3, MARCH5, UHRF1, and ZFP91 (Table S3).

Importantly, none of these 10 genes were identified in four previous genome-wide siRNA screens performed in cell lines to identify host factors affecting HIV infection (8, 36–38). This discrepancy may be due in part to differences in our methodologies, from the approach to genetic manipulation (CRISPR-Cas9 gene knockout vs. siRNA/shRNA knockdown), the delivery (electroporation vs. transfection/transduction), the cell types used (primary CD4+ T cells vs. cell lines), and/or the scope of the study (E3s detected by proteomics vs. genome-wide). Importantly, this is the first time that 7 of these 10 E3 ligases (all but ZFP91, UHRF1, and VPS18) have been reported to functionally affect HIV infection in any cell system and the first time that all 10 have been shown to affect HIV replication in a relevant model of infection: primary CD4+ T cells.

### Network propagation analysis connects TRAF2, non-canonical NF-κB signaling, and HIV infection

**Fig 3:**
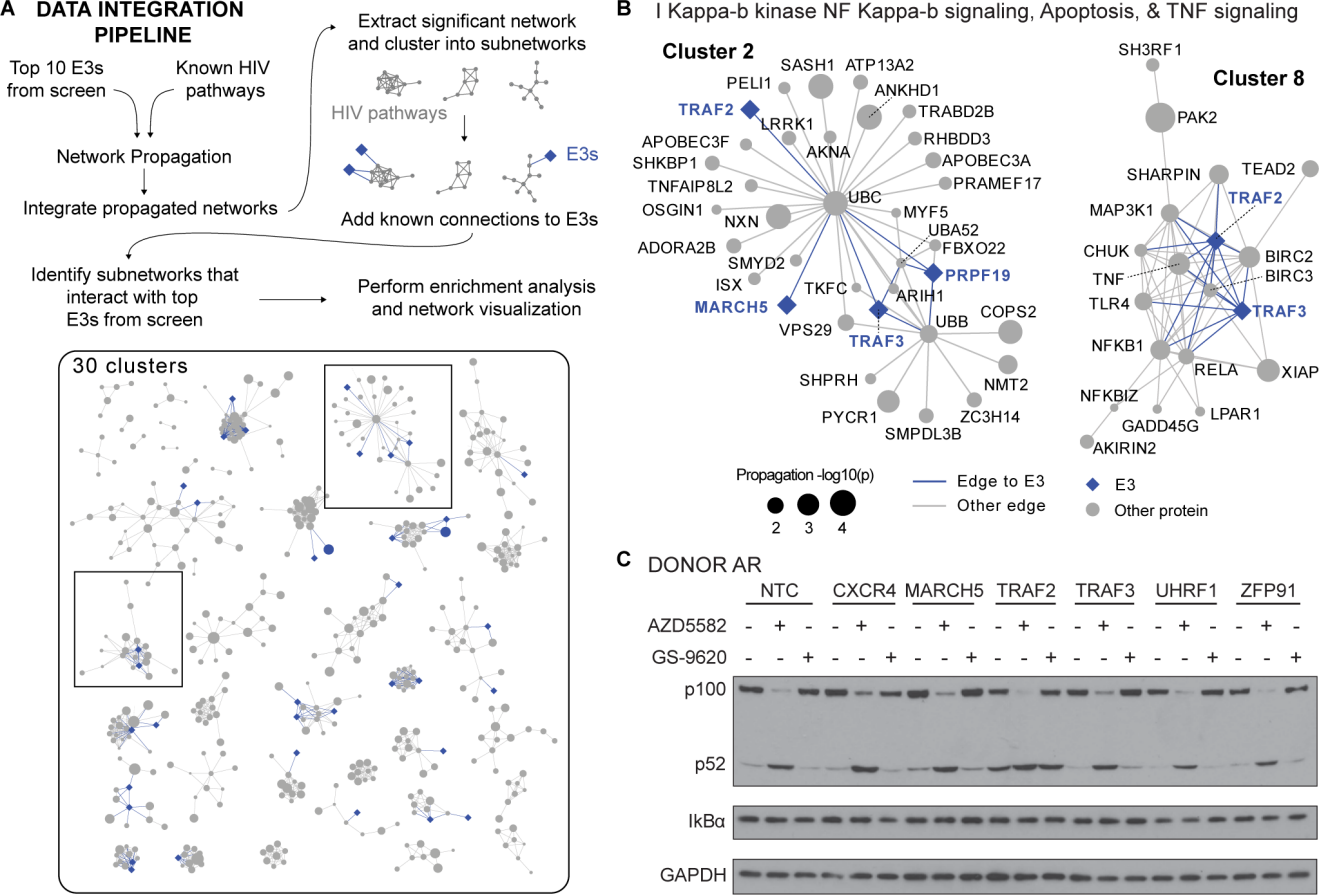
Network propagation analysis connects TRAF2, non-canonical NF-κB signaling, and HIV infection. (A) Data integration pipeline overview. The top E3 hits from this study and human proteins with prior association with HIV were integrated using network propagation. The significant subnetwork from the propagation was further clustered into 30 subnetwork pieces (or “clusters”), and known functional and physical connections between each cluster and the top E3s from this study were added. This analysis serves to highlight the pathways and complexes that are associated with both HIV pathogenesis and the E3s from this study. (**B**) Cluster 8 (C8) extracted from the network propagation results possesses significant enrichment in NF-κB-related pathway terms. E3s from this study are annotated in blue diamonds. Gray circles denote human proteins within the base network. The size of the circle denotes the −log10(*P* value) of the propagation result. (**C**) Western blot analysis probing for non-canonical NF-κB activation (marked by the processing of p100 to p52) and non-canonical NF-κB activation (marked by IкBα degradation), with a GAPDH loading control in gene knockouts in primary activated CD4+ T cells from healthy human donor AR, which is one representative donor of three (Fig. S3).

To assess connections between our top 10 E3s more systematically and identify the pathways hijacked by HIV, we performed a network propagation analysis (39). This analysis is used to understand how genes of interest are interconnected within large biological networks that capture known functional and physical interaction information. Here, we independently propagated (1) human proteins associated with HIV extracted from pathway databases and (2) the top 10 E3s from this study across a large network comprised of ReactomeFI (40), CORUM (41), and known HIV-host complexes (11). We integrated the propagated networks gene-wise by multiplication and performed a permutation test to identify and extract the significant subnetwork, which we clustered into 30 smaller subnetworks (Table S4). We then manually annotated clusters connected to the 10 E3s identified in our screen (Fig. 3A).

Of the 30 HIV pathway subnetworks we uncovered, 19 contained at least one of the 10 E3s from our knockout screen in HIV-infected CD4+ T cells, including four of the five higher-confidence hits: TRAF2 (11 clusters), TRAF3 (9 clusters), ZFP91 (2 clusters), and MARCH5 (1 cluster) (Fig. S2; Table S4). Expectedly, TRAF2 and TRAF3 overlapped in nine clusters, five of which had connections to NF-κB signaling and function, including tumor necrosis factor-mediated signaling which is an upstream activator of NF-κB, activation of innate immune response (which represents multiple pathways including NF-κB), and NIK NF-κB signaling which is involved in the activation of the non-canonical NF-κB pathway. Consistent with these analyses, TRAF2 and TRAF3 are non-canonical NF-κB inhibitors that function together in a complex with BIRC2 and BIRC3, a connection captured in cluster 8 (Fig. 3B). This complex is also important for HIV latency reversal, as BIRC2 inhibition by AZD5582 was recently shown to increase transcription of HIV mRNA and reverse latency (42, 43). Cluster 8 also contains NFKB1 and RELA, both transcription factors that bind the *NF-κB* promoter in canonical NF-κB signaling.

Since our analysis and previous studies (44, 45) connected TRAF2 and TRAF3 to NF-κB signaling, we tested the effect of each gene knockout for its ability to activate canonical and non-canonical NF-κB alone or in combination with two latency-reversing agents (LRAs), AZD5582 and GS-9620. For comparison, three other high-confidence E3 ligases (MARCH5, ZFP91, and UHRF1) along with CXCR4 and NTCs were also included in the test. Western blot analysis was used to monitor non-canonical NF-κB activation (processing of p100 to p52) and canonical NF-κB activation (degradation of IкBα). As expected, treatment with AZD5582 increased p52 levels in all cells tested whereas TLR7 agonist GS-9620 did not, a result that was anticipated as the latter drug is proposed to work only in dendritic cells (46) (Fig. 3C; Fig. S3). Deletion of TRAF2, but not the other genes tested, resulted in increased p52 levels, demonstrating that it is a regulator of non-canonical NF-κB signaling (Fig. 3C; Fig. S3). Surprisingly, TRAF3 did not share this phenotype, possibly owing to the fact that TRAF2 could be playing a role independent of TRAF3 and/or incomplete genetic ablation of TRAF3. More work will be required to understand the functional differences between TRAF2 and TRAF3 in these and other assays connected to HIV infection.

Interestingly, our analysis revealed several other connections between the E3 ligases uncovered in our screen and the clusters related to HIV function defined by network propagation. For example, the splicing factor PRPF19 was present in 10 clusters enriched for roles in TNF signaling, NF-κB signaling, transcription, and DNA repair (Fig. S4). Also, PPIL2, a cyclophilin family member, was found in clusters C17 and C26, which are linked to transcription and mRNA processing (Fig. S4). The nuclear factors RING1 and ZFP91 were enriched in clusters related to nuclear transport (C5) and transcription (C6) (Fig. S4). Further studies will aid in understanding these and many of the other connections revealed by the network propagation analysis.

### Deletion of TRAF2 and UHRF1 reverses HIV latency in different JLat cell line models

**Fig 4:**
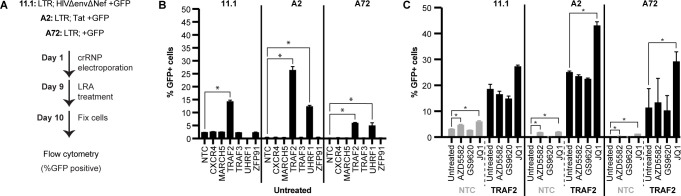
TRAF2 and UHRF1 knockouts reverse latency in JLat models. (A) Workflow describing gene knockout and treatment with LRAs in JLat cell lines 11.1, A2, and A72. (**B**) Flow cytometry quantification of latency reversal (% GFP+ cells) for gene knockouts that were untreated (no LRAs) in JLat cell lines 11.1, A2, and A72. One sample that did not pass the viability cutoff of 30% lymphocytes was removed and denoted “NA.” Significance was defined as a fold change ≥ 1.5 and a *P* value <0.05 compared with the untreated NTC within the same cell line. (**C**) Flow cytometry quantification of latency reversal (% GFP+ cells) for NTCs and TRAF2 knockouts that were untreated or treated with the LRAs AZD5582, GS-9620, or JQ1 in JLat cell lines 11.1, A2, and A72. Significance was defined as a fold change ≥ 1.5 and a *P* value <0.05 compared with the untreated condition of the same gene knockout within the same cell line. Significance was defined as a fold change ≥ 1.5 and a *P* value <0.05 compared with NTC within the same donor.

While only TRAF2 was found to regulate non-canonical NF-κB signaling, the other four high-confidence E3s were still connected to pathways related to latency regulation and reversal, such as viral transcription, epigenetic regulation, and LRAs. To test whether our five high-confidence E3s regulate HIV latency reversal, we deleted each gene in Jurkat cell models of latency (JLats) using CRISPR. We used three JLat cell lines that have an integrated HIV promoter and a GFP reporter with low baseline expression, including 11.1s (LTR-full length HIVΔenvΔNef-GFP), A2s (LTR-Tat-GFP), and A72s (LTR-GFP)(47). TRAF2, TRAF3, UHRF1, ZFP91, and MARCH5 were deleted individually in each JLat cell line by electroporation with pre-formed crRNPs using the guide that displayed the strongest phenotype in our previous screen. After 8 days, HIV was reactivated using TNF, a potent LRA, for 24 hours, and then, latency reversal was analyzed by flow cytometry (Fig. 4A). The percentage of GFP+ cells was measured and compared with not only NTCs but also the deletion of CXCR4, since its role in HIV entry is not relevant to JLats and should not affect latency reversal.

As expected, the percentage of untreated NTC cells expressing GFP was very low in all three cell lines (Fig. 4B; Table S5). Compared with untreated cells, treatment with TNF induced GFP expression in 11.1 cells by 19-fold, in A2s by 109-fold, and in A72s by 56-fold (Fig. S5B; Table S5). In comparison to untreated NTC cells, deletion of TRAF2 increased GFP expression in 11.1, A2, and A72 cell lines 6-fold, 59-fold, and 27-fold, respectively (Fig. 4B; Table S5). While TRAF2 knockout was not toxic (Fig. S5A; Table S5), the addition of TNF to 11.1 cells resulted in samples that fell below our 30% viability threshold (Fig. S5A; Table S5). However, TNF-treated TRAF2 knockouts in A2s and A72s synergistically increased GFP expression 1.5-fold and 2.3-fold, respectively, compared with TNF-treated NTCs (Fig. S5B; Table S5).

While UHRF1 deletion in 11.1 cells resulted in less than 30% viability (Fig. S5A; Table S5), deletion in A2s and A72s increased GFP expression 27.4-fold and 22.6-fold, respectively (Fig. 4B; Table S5), consistent with recent data connecting UHRF1 to HIV latency reversal (48). Deletion of either CXCR4, TRAF3, ZFP91, or MARCH5 did not increase GFP expression in any of the JLats used (Fig. 4B; Fig. S5).

To interrogate if either TRAF2 and UHRF1 knockouts could work synergistically with LRAs for latency reversal, we treated these TRAF2- and UHRF1-deleted cells with a panel of LRAs, including JQ1 (p-TEFb activator), AZD5582 (inhibitor of apoptosis proteins and a SMAC mimetic), and GS-9620 (TLR7 agonist) (49, 50). Importantly, JQ1 and AZD5582 induced GFP expression in JLats whereas TLR7 agonist GS-9620 did not, presumably because the TLR7 pathway is not active in these cells (46). Unfortunately, deletion of UHRF1 combined with any of the LRAs resulted in toxicity in all three JLat model cells. However, treatment of TRAF2 knockouts with JQ1 increased GFP expression compared with the corresponding untreated cells, suggesting they may target different latency-reversing pathways (Fig. 4C; Table S5) (51). In contrast, the combination of AZD5582 with deletion of TRAF2 did not result in a detectable increase in GFP expression (Fig. 4C). This could be due to either the very modest reactivation of HIV-1 latency by AZD5582 or a potential epistatic relationship that could suggest that TRAF2 works in the same pathway that AZD5582 targets.

### Deletion of TRAF2 and UHRF1 reverses HIV latency in a novel CRISPR-compatible resting primary human CD4+ T cell model of latency

**Fig 5:**
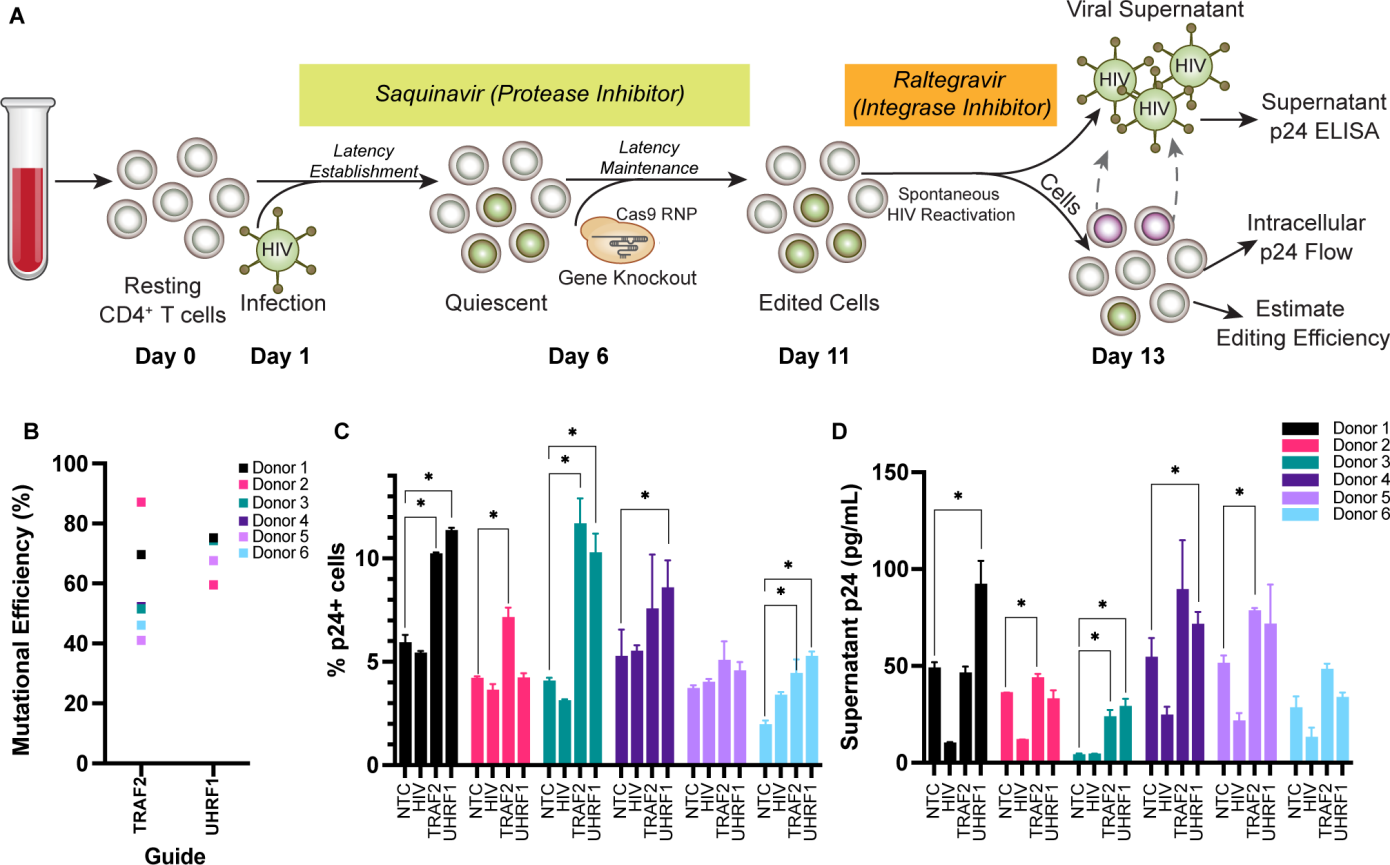
TRAF2 and UHRF1 knockout reverse latency resting primary human CD4+ T cells. (A) Workflow describing gene knockout and spontaneous HIV reactivation in a novel CRISPR-compatible primary resting CD4+ T cell latency model. (**B**) Quantification of TRAF2 and UHRF1 knockouts by sequencing. Sanger sequencing was analyzed for mutational efficiency by TIDE (59). (**C**) Flow cytometry quantification of latency reversal (% p24+ cells) for gene knockouts in primary resting CD4+ T cells. (**D**) ELISA quantification of latency reversal (supernatant p24 [pg/mL]) for gene knockouts in primary resting CD4+ T cells from six healthy human donors. Significance was defined as *P* value <0.05 compared with NTC within the same donor.

The establishment and regulation of HIV latency can be affected by both virus-related and host-cell-related factors. Immortalized cell lines have given invaluable insights into the regulation of HIV latency given their tractability for genetic manipulation and chemical screening. However, these highly activated cell line models make use of replication-incompetent viruses, are clonal, and have altered functional states and signaling pathways as compared with physiologically relevant primary resting CD4+ T cells. The fact that several LRAs activate T cells to reverse latency indicates that the cell activation state is an important variable in latency regulation (52) and potential hits should be validated in resting primary cells (53, 54). Therefore, we have developed a novel CRISPR-compatible resting T-cell latency model (CREST-L) for testing HIV latency directly in resting primary human CD4+ T cells and used it to validate the latency reversal potential of TRAF2 and UHRF1 (Fig. 5). CREST-L does not require T cell activation and thus avoids the combined confounding effects resulting from activation-induced cell death and activation-induced HIV reactivation. To date, HIV infection of resting T cells has been inefficient, and most primary cell models of latency circumvent this problem by using spinoculation to directly infect resting CD4+ T cells (55, 56). Though spinoculation achieves higher HIV infection rates in resting T cells compared with regular infection, we optimized this procedure further to improve the yield and throughput of our assay. For this, we included low levels of IL-7 in our culture medium since this cytokine has been previously shown to improve the survival of HIV-infected resting T cells without activating them or disrupting HIV latency (57). Moreover, we have previously shown that IL-7 treatment can improve both gene-knockout and knock-in efficiencies in activated primary human immune cells (58). To assess whether IL-7 treatment can increase the rate of HIV infection in resting primary human CD4+ T cells, we cultured the cells overnight in complete RPMI media with very low levels of IL-2 (10 IU/mL) and IL-7 (5 ng/mL), and then, the cells were infected with a replication-competent HIV-1 virus strain (HIV-LAI.2 WT, Subtype B, CXCR4 tropic) by spinoculation. Intracellular p24 staining measured by flow cytometry (Fig. S6B) showed increased p24+ cells in IL-7-treated samples as compared with IL-7-untreated samples, increasing HIV infection by up to 3.6-fold (Fig. S6C).

After optimizing HIV infection in resting CD4+ T cells, we adapted our RNP-based CRISPR gene-editing approach for achieving efficient editing in resting T cells. We have previously found that stabilization of Cas9 RNPs with non-homologous single-strand oligo (ssODNenh) or with polyglutamic acid (PGA) can significantly improve editing efficiency in diverse primary cell types post-activation, such as bulk (CD3+) T cells, CD8+ T cells, CD4+ T cells, and regulatory T cells (Tregs) (60). In order to identify the best conditions for resting T cell editing, we isolated resting CD4+ T cells from two healthy human donors by negative selection, rested them overnight, and electroporated the next day with crRNPs using either a non-targeting control guide (NTC-03; Table S2) or one of the two TRAF2 guides resulting in the strongest phenotypes in activated CD4+ T cells in our previous screen (TRAF2-01 and TRAF2-03; Table S2). Each crRNP was complexed with either ssODenh or PGA. Three days following electroporation, cells were lysed, genomic DNA was isolated, and the region around the cut sites was amplified by PCR for estimation of editing efficiency by tracking of Indels by DEcomposition (TIDE) analysis (59). In resting human CD4+ T cells, we found ssODenh to be a superior electroporation enhancer for editing. The inclusion of ssODenh improved editing efficiency for two TRAF2 guides by up to fourfold as compared with PGA (Fig. S6D). More importantly, the improvement in editing efficiency was more pronounced for guides with poor editing efficiency making this approach particularly useful for knocking out those genes for which efficient guides are unavailable.

The next step was to combine HIV infection of resting CD4+ T cells with CRISPR gene editing and test the latency reversal potential of TRAF2 and UHRF1 directly in primary resting CD4+ T cells. The combined workflow is as follows (Fig. 5A): resting CD4+ T cells were isolated from six independent healthy human donors by negative selection, rested for 24 hours, and then infected with a replication-competent HIV-1 virus strain (HIV-LAI.2 WT, Subtype B, CXCR4 tropic) by spinoculation in technical triplicate. Cells were immediately cultured in the presence of Saquinavir, a protease inhibitor that prevents the formation and release of mature virions, allowing the prevention of spreading infection (54, 55). Six days following infection, the cells were electroporated with crRNPs in technical triplicate using either a non-targeting control guide (NTC-03; Table S2) or the TRAF2/UHRF1 guide resulting in the strongest phenotype in our previous screen (TRAF2-01 and UHRF1-01; Table S2). As an additional control, we included crRNPs containing two HIV-targeting guides (HIV-GAG-01 and HIV-GAG-02). Directly targeting integrated proviruses with RNPs should result in less virus production from infected cells. The HIV infection rate was between 17.0% and 30.9% in all six donors on the day of electroporation (Table S6). Cells were incubated with Saquinavir for 5 more days, which was then replaced by fresh media containing Raltegravir, an integrase inhibitor. Removal of Saquinavir allows successful production of mature virions from latently infected cells, while Raltegravir inhibits integration of any newly produced viral particles, thereby preventing the viral spread and allowing an accurate estimation of the latency reversal (54, 55). After culture for 2 days in the presence of Raltegravir, cells and supernatant were collected for intracellular staining of HIV p24 or p24 ELISA since intracellular p24 levels reflect latency reversal at the level of HIV transcription and translation, while p24 levels in the supernatant measure the assembly and release of virions. We achieved 41% to 91.3% knockout efficiency for TRAF2 and 59.5% to 75.2% editing efficiency for UHRF1 in resting CD4+ T cells from these six donors, determined by TIDE analysis (Fig. 5B). Cell viability was more than 40% for all KO and donors (Fig. S6E).

Intracellular p24 staining measured by flow cytometry showed significantly increased p24+ cells in TRAF2 knockouts compared with NTC in five out of six donors, increasing by 1.9-fold on average (Fig. 5C). UHRF1 KO also resulted in an increase in intracellular p24 staining in five out of six donors, increasing by twofold on average (Fig. 5C). The increase in intracellular p24 levels was statistically significant in four out of six donors for both TRAF2 and UHRF1. These results suggest that both TRAF2 and UHRF1 play a role in HIV latency and are involved in regulating HIV transcription. Blocks to HIV production from infected cells can be present at multiple levels, and therefore, removing a block from HIV transcription and translation might not necessarily result in HIV production from latently infected cells. Therefore, we also measured HIV production in culture supernatants by p24 ELISA and found an increase in HIV production with TRAF2 knockouts compared with NTC in five of six donors (statistically significant in three donors, fold change > 1.5 and *P* value >0.05) (Fig. 5D; Table S6). Similarly, p24 levels in supernatants were higher with UHRF1 knockouts compared with NTC in four out of six donors, increasing by 2.6-fold on average (Fig. 5D; Table S6). Cells treated with HIV-targeting control crRNPs showed a significant decrease in HIV production demonstrating the expected results of editing on virus production (Fig. 5D). Cells from donors 4, 5, and 6 were also electroporated with a safe-harbor site (AAVS1) targeting crRNPs and two additional non-targeting control crRNPs. As expected, cells treated with these additional controls showed supernatant p24 levels comparable to that of cells treated with the original NTC crRNP control further demonstrating the specificity of increased HIV production seen with TRAF2 and UHRF1 knockouts (Fig. S6F).

## DISCUSSION

In this study, we have investigated the role of E3 ubiquitin ligases in HIV infection and latency directly in primary human CD4+ T cells. Ubiquitin signaling plays a pivotal role in regulating essential cell processes, including apoptosis and cell cycle, that are perturbed in immortalized cell lines. In addition, the components of the ubiquitin proteasome system play a vital role during HIV infection in cells, both in promoting replication and in overcoming innate immune defenses (10). For example, during HIV infection, it has been shown that viral proteins hijack E3 ligases and the ubiquitin proteasome system to target host factors to reduce superinfection (i.e., CD4 and CXCR4) (61), increase viral transcription (i.e., IκBα) (62), promote viral infectivity (i.e., SERINC5, PSGL-1, SMUG, and UNG2)(61, 63–65), and inhibit innate immune response (APOBEC3G/F/H, Tetherin) (66, 67). Therefore, we decided to probe the role of E3 ligases directly in primary human T cells and identified therapeutically valuable targets for HIV infection as well as latency reversal. To accomplish this, we first identified proteins expressed in primary CD4+ T cells from three healthy donors by mass spectrometry and found that 116 single-subunit E3s were expressed in at least one donor. Using our recently developed arrayed CRISPR-Cas9 knockout screening pipeline for studying HIV-host interactions in primary CD4+ T cells (26, 27), we systematically deleted 116 expressed E3s (and related family members). We found that 10 had a significant impact on HIV infection both positively (three anti-viral: TRAF2, TRAF3, and PRPF19) and negatively (seven pro-viral: MARCH5, ZFP91, UHRF1, VPS18, NOSIP, PPIL2, and RING1). We did not individually test for knockout efficiency of the 116 targets, leaving the possibility to miss host genes that could play a role in HIV infection. Importantly, three significant genes (UHRF1, ZFP91, and VPS18) had previously published roles in HIV infection. Previous systematic genetic screens of HIV host factors in cell lines (8, 36–38) did not overlap with any of our 10 E3 hits, emphasizing the importance of using physiologically relevant primary CD4+ T cells, the *in vivo* target of HIV. In summary, the data presented here increased the pool of potential E3 Ub ligase targets that could be utilized in the development of host-directed therapies.

To better understand the role of these E3s in HIV infection, we employed network propagation (39) using our data and known HIV data sets to identify different players linked to NF-κB signaling. This analysis helps to understand how genes of interest are interconnected within extensive biological networks that incorporate known functional and physical interactions. We found that TRAF2 is a key regulator of non-canonical NF-κB signaling in primary T cells. Consistent with this, previous work has shown other proteins that function in the same pathway as TRAF2, notably BIRC2 and BIRC3, which are also involved in regulating NF-κB signaling and HIV transcription and latency (42, 68, 69). Furthermore, TRAF2, TRAF3, and other components of the NF-κB signaling pathway have been implicated in regulating viral infection and latency programs of other viruses, including Kaposi’s sarcoma herpesvirus (KSHV) (70, 71), herpes simplex virus type 2 (HSV-2) (72), Epstein-Barr virus (EBV) (73–75), and Human T-cell leukemia virus type 1 (HTLV-1) (76, 77). UHRF1 has also been implicated in EBV latency (78) and in regulating HIV-1 transcription and latency (48, 79). Given the critical role of the BIRC2/3 complex in HIV latency reversal (42), the role of some of our hits in viral latency, and the fact that HIV latency is the main barrier for HIV cure, we chose to further probe the role of several E3 ligases (TRAF2, TRAF3, MARCH5, ZFP91, and UHRF1) specifically in the context of HIV latency. We found that deletion of either TRAF2 or UHRF1 resulted in the reversal of HIV latency in multiple JLat models. Specifically, the deletions resulted in spontaneous reactivation of latent HIV transcription and did not require treatment with LRAs. Treatment of gene knockouts with LRAs informed pathways of action, as TRAF2 deletion treated with TNFα (canonical NF-κB activator) and JQ1 (p-TEFb activator) was synergistic, suggesting that TRAF2 functions in different pathways as expected and could represent a therapeutic target for combined treatments (80).

To assess latency reversal in a more physiologically relevant system, we developed a novel CRISPR-compatible latency reversal assay in resting primary human CD4+ T cells (CREST-L). This model is based on widely used Greene and O’Doherty primary cell models of latency (55, 56). CREST-L overcomes previous limitations of modeling latency in resting T cells by achieving the high levels of HIV infection necessary to examine latency reversal phenotypes without a need for cell activation in resting CD4+ T cells, an important but previously less tractable cell population. Through extensive optimization experiments, we also found that addition of ssODenh electroporation enhancer to standard CRISPR-Cas9 crRNPs significantly improves editing efficiency in resting T cells without changes to buffer or electroporation conditions. With these improvements, our resting T cell editing method will be widely useful for additional applications beyond studies of HIV infection and latency. While the JLat models of latency used in this study are based on Env-deleted proviruses and therefore only report HIV transcription and translation, CREST-L allows us to probe both HIV transcription and translation as well as virion production since it makes use of a fully replication-competent HIV. It also recapitulates the multiple integration sites found across latently infected cells, while clonal JLat models have only a single unique integration site. Furthermore, the signaling pathways and molecules regulating latency reversal, especially ubiquitin pathways, are closer to physiological conditions in the primary cell model than in JLat cell lines. Taken together, the technique established in this study represents an important advancement for the ability to edit resting primary human T cells, which we hope will be useful across fields.

In the current study, our CRISPR-compatible primary cell model of HIV latency revealed that TRAF2 deletion increased intracellular p24 in all six donors and UHRF1 deletion increased intracellular p24 in four of six donors. These results are consistent with the phenotypes observed in JLat cells and suggest transcriptional and translational reactivation of latent HIV-1 after gene deletion. Strikingly, TRAF2 deletion also increased supernatant p24 (virus production) in five of six donors, while UHRF1 deletion increased supernatant p24 in four of six donors, suggesting the reactivation of full-length viruses. Importantly, in this system, we confirm that for both intracellular and supernatant p24 data, TRAF2 and UHRF1 depletion alone reversed latency without any additional treatment with LRAs.

The mechanism by which TRAF2 knockouts increase HIV infection and reverse latency requires further investigation. However, we have proposed a model based on our literature review and observation of non-canonical NF-κB activation by western blot after TRAF2 depletion. The NIK regulatory complex comprises TRAF2, TRAF3, BIRC2, and BIRC3 and functions to suppress non-canonical NF-κB signaling. TRAF2 binds BIRC2 and BIRC3, in which K48 ubiquitinates NIK to lead to its degradation. TRAF2 also binds TRAF3, which binds NIK to bring it into proximity for ubiquitination by BIRC2 and BIRC3. When NIK is degraded, the non-canonical NF-κB pathway is inactive. However, upon TRAF2 knockout, BIRC2 and BIRC3 are no longer in proximity to NIK for K48 ubiquitination, and NIK is allowed to accumulate. NIK then phosphorylates Ikkα, which phosphorylates p100. p100 is normally sequestered in the cytoplasm. However, upon phosphorylation, it is recognized by the SCF^βTrCP^ ubiquitin ligase complex and processed into p52. p52 in complex with RELB can then translocate to the nucleus and bind the HIV LTR to promote transcription of viral mRNA. Increased viral transcription leads to increased active HIV infection (seen in our screen) and latency reversal (as seen in JLat and primary cell models).

However, our evidence suggests that TRAF2 is likely affecting more pathways than just non-canonical NF-κB. For example, the combination of TRAF2 knockout and LRA treatment with BIRC2 inhibitor AZD5582 did not reverse latency in JLats nearly to the same extent as with TRAF2 knockout alone. BIRC2 and TRAF2 are members of the same NIK regulatory complex, yet there is no observed synergistic effect of combined TRAF2 knockout and BIRC2 inhibition. Elucidating additional pathways by which TRAF2 reverses HIV latency will be important for the development of novel LRAs that take advantage of TRAF2-mediated signaling pathways and regulation of HIV latency reversal.

**Fig 6:**
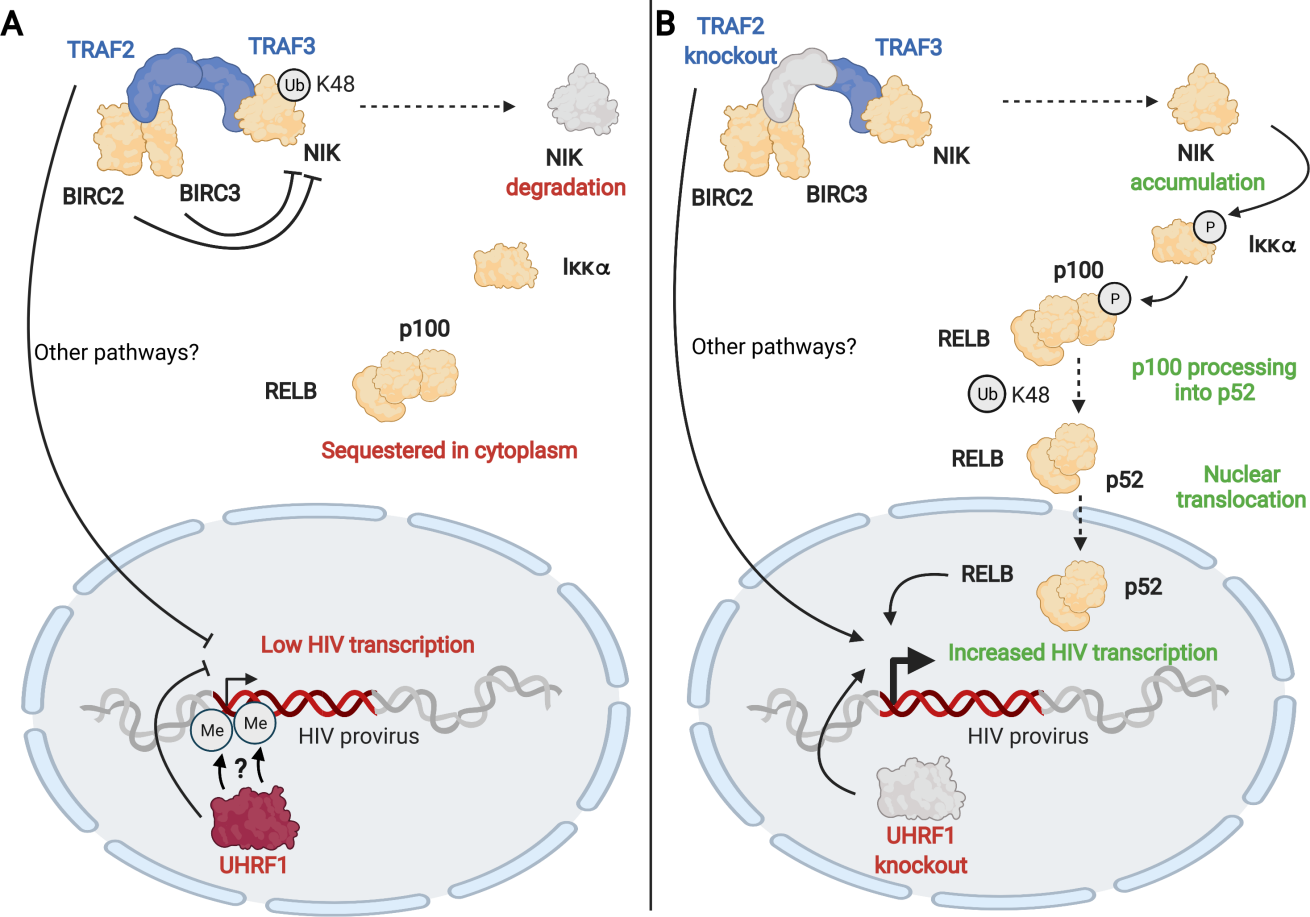
Proposed model by which TRAF2 and UHRF1 transcriptionally reactivate the latent HIV-1 promoter. (A) Low HIV-1 transcription in the presence of TRAF2 and UHRF1. (**B**) Increased HIV-1 transcription upon TRAF2 knockout and UHRF1 knockout. Created with BioRender.com.

In addition to TRAF2, we found that UHRF1 is another key regulator of HIV infection and HIV latency. UHRF1 deletion decreased active infection in primary activated CD4+ T cells and increased latency reversal in JLat and primary resting CD4+ T cell models. We hypothesize that UHRF1 may control HIV-1 transcription based on a recent publication that suggests a role for it in the epigenetic regulation of HIV-1 (81) (Fig. 6). UHRF1 may recruit DNA methyltransferases to silence the HIV-1 promoter by DNA methylation, resulting in repressed HIV transcription and maintaining HIV latency. In this recent study, knockdown of UHRF1 in JLat models and drug inhibition of UHRF1 in *ex vivo* HIV+ patient samples reactivated HIV-1 transcription and virion production. Consistent with this, we have observed that UHRF1 knockout increased HIV-1 transcription in JLat models and increased both transcription and virion production in a resting primary human CD4+ T cell model of latency.

Interestingly, in our study, UHRF1 deletion decreased active infection but increased latency reversal. This suggests two independent roles for UHRF1 at different stages of HIV infection, where UHRF1 is required for the establishment of initial infection but then hinders HIV-1 production and contributes to the maintenance of latency. Both roles can be considered pro-viral, as UHRF1 is needed for effective initial infection, and its maintenance of latency aids long-term infection in the host. While a potential mechanism for UHRF1 in latency maintenance has been recently described (81), future work is needed to identify a mechanistic role for UHRF1 in initial HIV infection.

Altogether, we have comprehensively studied E3 ligases expressed in primary human CD4+ T cells for a functional role in HIV infection and identified 10 E3s enriched for roles in NF-κB regulation (Fig. S7), two of which also have a functional role in HIV latency reversal. We have developed a novel assay for studying the impact of gene deletions on latency reversal in resting primary human CD4+ T cells that is of use to the broader HIV research community. Deletion of TRAF2 and UHRF1 reverses latency in JLat and primary cell models, making them excellent candidates for the development of novel LRAs, as well as a further mechanistic study into their role in HIV infection and latency.

## MATERIALS AND METHODS

### Cell lines

Jurkat (JLat) cell lines 11.1, A2, and A72 (47) were cultured in complete Roswell Park Memorial Institute (RPMI) media, consisting of RPMI-1640 media (Corning) with 10% FBS (Gibco), 50 µg/mL penicillin-streptomycin (Fisher), and 2 mM L-glutamine (Corning) at 37°C/5% CO_2_/humid/dark. JLat cell lines were a generous gift from Melanie Ott’s lab.

The HEK293T cell line was cultured in complete Dulbecco’s modified Eagle medium (DMEM) media consisting of DMEM media (Corning) with 10% FBS (Gibco) and 50 µg/mL penicillin-streptomycin (Corning) at 37°C/5% CO_2_/humid/dark.

### Isolation and activation of primary CD4+ T cells

CD4+ T cells were isolated from healthy human donors using residuals from leukoreduction chambers after Trima Apheresis (Blood Centers of the Pacific, Vitalant). By positive selection, CD4+ T cells were isolated by the FABian cell isolation system (Cellcopedia) and IBA Lifesciences CD4 isolation kit for FABian (IBA Lifesciences, Fisher Scientific). By negative selection, peripheral blood mononuclear cells were isolated by Ficoll centrifugation using SepMate tubes (STEMCELL, per manufacturer’s instructions). PBMCs were used fresh or frozen at 250 million cells in 1 mL of 90% FBS 10% DMSO and stored in liquid nitrogen. CD4+ T cells were isolated from PBMCs by magnetic negative selection using an EasySep Human CD4+ T Cell Isolation Kit (STEMCELL, per manufacturer’s instructions). Isolated CD4+ T cells were activated for 72 hours by plate-bound CD3 (Tonbo Biosciences clone UCHT1) (150 µL of 10 µg/mL CD3 in PBS in non-TC-treated 48-well plates, incubated for at least 12 hours at 4°C, and then aspirated before adding cells) and 5 µg/mL CD28 (Tonbo Biosciences clone CD28.2) in complete RPMI media, consisting of RPMI-1640 media (Corning) with 10% FBS (Gibco), 50 µg/mL penicillin-streptomycin (Corning), 5 mM sodium pyruvate (Corning), 5 mM 4-(2-hydroxyethyl)-1-piperazineethanesulfonic acid (HEPES) (HyClone), and 20 IU/mL IL-2 (Miltenyi Biotec) at 37°C/5% CO_2_/humid/dark.

### Sample preparation for global abundance proteomic analysis of primary CD4+ T cells

Primary CD4+ T cells from three healthy human donors were activated by anti-CD2/anti-CD3/anti-CD28 beads (Miltenyi Biotech T cell activation/expansion kit, human) at a 1:1 bead:cell ratio and cultured in complete media [RPMI-1640 media (Corning) with 10% FBS (Gibco), 50 μg/mL penicillin-streptomycin (Corning), 5 mM sodium pyruvate (Corning), 5 mM 4-(2-hydroxyethyl)-1-piperazineethanesulfonic acid (HEPES) (HyClone), and 20 IU/mL IL-2 (Miltenyi Biotec)] at 37°C/5% CO_2_/humid/dark for 6 days. Cells were pelleted by centrifugation at 400 *× g* for 5 min and washed three times with PBS. After the last wash, cell pellets were resuspended in 100 µL of lysis buffer (8 M urea, 0.1 M ABC pH 8, 150 mM NaCl, 1 mini-cOmplete protease inhibitor, and 1 PhosSTOP phosphatase inhibitor brought to 10 mL with H_2_O) and frozen at −80°C. Samples were thawed on ice and lysed by probe sonication in three pulses of 20% amplitude for 15 s. Lysates were clarified by centrifugation at 16,100 × *g* at 4°C for 30 min. Protein concentration was measured by Bradford assay (Thermo), and 0.16 mg of total protein was reduced with 4 mM tris(2-carboxyethyl)phosphine for 30 min at room temperature and alkylated with 10 mM iodoacetamide for 30 min at room temperature in the dark. Excess iodoacetamide was quenched with 10 mM 1,4-dithiothreitol for 30 min at room temperature in the dark. The samples were diluted fourfold in 100 mM ammonium bicarbonate, pH 8.0, to a final urea concentration of 2 M. Samples were incubated with 4 µg of sequencing grade modified trypsin (Promega) and incubated at room temperature with rotation for 18 h. The digests were acidified by the addition of 10% trifluoroacetic acid (TFA) to a final concentration of 0.3% trifluoroacetic acid (pH ~2). Insoluble material was removed by centrifugation at 16,000 *× g* for 10 min. Peptides were desalted using UltraMicroSpin C18 columns (The Nest Group). The columns were activated with 200 µL of 80% acetonitrile (ACN) and 0.1% TFA and equilibrated three times with 200 µL of 0.1% TFA. Peptide samples were applied to the columns, and the columns were washed three times with 200 µL of 0.1% TFA. Peptides were eluted with 140 µL of 50% ACN and 0.25% formic acid and lyophilized. The samples were fractionated using the Pierce High pH Reversed-Phase Peptide Fractionation Kit (Thermo cat. no. 84828) according to the manufacturer’s instructions. Samples were dissolved in 0.1% TFA, bound to the column, and washed with water. Any peptides not binding to the column (flow-through) or eluted during the water wash (column wash) were also analyzed by MS. Eight fractions per sample were then eluted by a stepwise gradient of acetonitrile (5%, 7.5%, 10%, 12.5%, 15%, 17.5%, 20%, and 50% acetonitrile) in 0.1% triethylamine. All samples were lyophilized prior to MS analysis.

### Mass spectrometry analysis for global abundance proteomics of primary CD4+ T cells

Fractionated samples were resuspended in 4% formic acid and 4% acetonitrile solution, separated by reversed-phase HPLC using a Thermo Easy n1200 LC (Thermo Scientific) using an in-house packed integrafrit column (360 µm O.D. × 75 µm I.D.) packed with 25 cm of 1.8 µm Reprosil C18 particles (Dr. Maisch-GMBL). Mobile phase A consisted of 0.1% FA in water and mobile phase B consisted of 80% acetonitrile (ACN)/0.1% FA. Peptides were separated at a flow rate of 300 nL/min by the following 2-h gradient: 8%–18% B over 52 min; 18%–38% B over 56 min; and 10 min at 88% B. Eluting peptides were analyzed by an Orbitrap Fusion Lumos Tribrid Mass Spectrometer (Thermo Scientific). Data were collected in positive ion mode with MS1 detection in profile mode in the orbitrap using 120,000 resolution, 350–1,350 m/z scan range, 25 ms maximum injection, and an AGC target of 5e5. MS2 fragmentation was performed on charge states from 2 to 5, MIPS mode = peptide, with a 40-s dynamic exclusion after a single selection, and 10 ppm ± mass tolerance. MS2 data were collected in centroid mode at a turbo scan rate in the ion trap with HCD (32% normalized collision energy), 15 ms maximum injection time, 2e4 AGC, 0.7 *m*/*z*. quadrupole isolation window, and 120 *m*/*z* first mass.

All raw MS data were searched with MaxQuant (v 1.6.2.6) against the human proteome (UniProt canonical protein sequences downloaded March 21, 2018). Peptides and proteins were filtered to 1% false discovery rate. MaxQuant default parameters were used with the exception that label-free quantification was turned on, with match between runs set to 0.7 min. .

### CRISPR-Cas9 RNP generation and electroporation in activated primary CD4+ T cells

Lyophilized tracrRNA (Horizon) and crRNA (all guide sequences designed by Horizon; Table S2) were resuspended at 160 µM in 10 mM Tris-HCL (7.4 pH) with 150 mM KCl. crRNPs were made by incubating 5 µL of 160 µM tracrRNA with 5 µL of 160 µM guide RNA and incubating for 30 min at 37°C; then, 10 µL of 40 µM Cas9 (Macrolab) was added and incubated for 15 min at 37°C. Stocks of crRNPs were plated in 3.5 µL aliquots in a 96-well plate and frozen for up to 6 months before use. Primary CD4+ T cells from two to three healthy human donors were ready for electroporation after 3 days of activation by plate-bound CD3 and in-solution CD28. Then, 3.5 µL of crRNPs was pipette mixed with 0.3e6-1e6 primary activated CD4+ T cells in 20 µL of Lonza electroporation buffer P3 and electroporated in Lonza 96-well nucleocuvette plates with code EH-115 using the Lonza 4D nucleofector core unit and Lonza 96-well shuttle device (Lonza). One hundred microliters of complete media [RPMI-1640 media (Corning) with 10% FBS (Gibco), 50 µg/mL penicillin-streptomycin (Corning), 5 mM sodium pyruvate (Corning), 5 mM 4-(2-hydroxyethyl)-1-piperazineethanesulfonic acid (HEPES) (HyClone), and 20 IU/mL IL-2 (Miltenyi Biotec)] was added immediately after electroporation, and cells were rested for 30 min at 37°C/5% CO_2_/humid/dark. Cells were then transferred to 96-well flat-bottom plates, brought to 200 µL with complete media, and cultured with anti-CD2/anti-CD3/anti-CD28 beads (Miltenyi Biotech T cell activation/expansion kit, human) at a 1:1 bead:cell ratio at 37°C/5% CO_2_/humid/dark. Cells were fed with complete media every 2–3 days until they were plated into replicates.

### Replica plating edited activated primary CD4+ T cells before HIV infection

Six days after electroporation, activated primary CD4+ T cells were plated into three technical replicates in 96-well U-bottom plates and brought up to 150 µL with complete media [RPMI-1640 media (Corning) with 10% FBS (Gibco), 50 μg/mL penicillin-streptomycin (Corning), 5 mM sodium pyruvate (Corning), 5 mM 4-(2-hydroxyethyl)-1-piperazineethanesulfonic acid (HEPES) (HyClone), and 20 IU/mL IL-2 (Miltenyi Biotec)]. Plates were “edgeless,” meaning that 200 µL of media was plated instead of the sample in rows A and H and columns 1 and 12, to prevent edge effects attributed to greater levels of evaporation. Experimental replica-plated cells were infected with HIV 1 day after cells were plated in technical triplicate. In addition to the experimental replica-plated cells, 2 × 60 µL of cells was collected and stored to analyze editing efficiency for each sample at the DNA and protein levels. Cellular DNA was collected from one sample by resuspending cells in QuickExtract DNA extraction solution (Lucigen) and incubating at 65°C for 20 min and then 95°C for 20 min. Protein samples were collected by resuspending cells in 2.5× Laemmli Sample Buffer (25 mM Tris pH 6.8, 8% glycerol, 0.8% SDS, 2% 2-mercaptoethanol, and 0.02% bromophenol blue) and incubation at 95°C for 20 min.

### HIV virion production

Fifteen-centimeter plates were seeded with 5e6 HEK293T cells in 25 mL complete media [DMEM media (Corning) with 10% FBS (Gibco) and 50 µg/mL penicillin-streptomycin (Corning)] 24 hours before transfection and cultured at 37°C/5% CO_2_/humid/dark. Per 15-cm plate, 10 µg of HIV-1 NL4-3 Nef:IRES:GFP or HIV-1 LAI.2 plasmid DNA was mixed with 250 µL serum-free DMEM media, and separately, 30 µL PolyJet DNA *In Vitro* Transfection Reagent (Signa Gen Laboratories) was mixed with 250 µL serum-free DMEM media (Corning). Plasmid DNA and PolyJet solutions were combined, pipette mixed twice, and incubated for 15 min at room temperature. The mixture was then added dropwise to seeded HEK293T cells in complete media and incubated in a BSL-3 setting at 37°C/5% CO_2_/humid/dark for 48 hours. Supernatants were collected and kept at 4°C, and cells were fed with 25 mL complete media and incubated at 37°C/5% CO_2_/humid/dark for 24 hours. After 24 hours, the second set of supernatants was collected and added to the previously collected supernatants. Combined supernatant was filtered through a 0.22-µm PVDF filter (Steriflip). For virus precipitation, 5.5 mL 50% PEG-6000 was mixed with 2.5 mL 4M NaCl, to which 24 mL of supernatant was added. Samples were inverted five times to mix and incubated 4–8 hours at 4°C. Precipitated virus was pelleted at 3,500 rpm for 20 min at 4°C and resuspended in 500 µL PBS. All prepared virus was combined and made into ≤1-mL aliquots in 2-mL micro tubes, flash frozen on dry ice, and stored at −80°C until use.

### HIV spreading infection in activated primary CD4+ T cells

Then, 2.5 µL of HIV-1 NL4-3 Nef:IRES:GFP was mixed with 47.5 µL complete media [RPMI-1640 media (Corning) with 10% FBS (Gibco), 50 µg/mL penicillin-streptomycin (Corning), 5 mM sodium pyruvate (Corning), 5 mM 4-(2-hydroxyethyl)−1-piperazineethanesulfonic acid (HEPES) (HyClone), and 20 IU/mL IL-2 (Miltenyi Biotec)] and added to replica-plated cells (150 µL of cells in complete media in 96-well U-bottom plates) to achieve ~2% infection after 48 hours. Plates were centrifuged at 1,200 × *g* for 2 hours at room temperature before incubating at 37°C/5% CO_2_/humid/dark. Two days after infection, 75 µL of sample was added to 75 µL 2% paraformaldehyde in PBS and the remaining samples were fed with 75 µL complete media. Four days after infection, 75 µL of sample was added to 75 µL 2% paraformaldehyde in PBS and the remaining samples were fed with 75 µL complete media. Six days after infection, 150 µL of sample was added to 50 µL 4% paraformaldehyde in PBS and any remaining sample was discarded.

### Flow cytometry and computational analysis of GFP-positive (HIV-infected) edited primary activated CD4+ T cells

Fixed cells were analyzed by an Attune NxT Acoustic Focusing Cytometer (Thermo Fisher), recording all events in a 100-µL sample volume after one 150-µL mixing cycle. Data were exported as FCS3.0 files and analyzed with a consistent template in FlowJo. Cells were gated on live lymphocytes, singlets, excluding autofluorescence, and then, the percentage of GFP+ cells was quantified (Fig. S6B). The data were exported to CSV, including the count and percentage of lymphocytes, the count and percentage of GFP+ cells, and the count and percentage of autofluorescent cells. The data were imported into R using RStudio and annotated with the sample information including donor, target gene, guide, days after infection, plate, and well. Individual wells with less than 21.8% lymphocytes or less than a count of 15,000 lymphocytes were excluded from analysis due to viability concerns. These represented the bottom 1% of the data set or 140 individual wells across all donors and guides (Fig. S1). Each well was normalized to the median of three non-targeting controls on its same plate for lymphocyte and GFP+ counts and percentages. After normalization, technical triplicates were averaged for the count and percentage of lymphocytes and the count and percentage of GFP+ cells and reported as a log2 fold change. The non-normalized averages for lymphocyte count and percentage and GFP+ count and percentage were also reported, and the data were exported as a CSV. For hit-calling, hits were defined as genes whose knockout yielded a log2 fold change in infection ≥ 1 or ≤−1 in the same direction in at least two time points for (i) two guides within the same donor or (ii) the same guide in two donors.

### Network propagation integrative analysis

We performed a network propagation-based analysis to identify pathways and protein complexes that converged between genes with known association with HIV pathogenesis and the top E3s identified in this study. Specifically, we used a heat-diffusion kernel analogous to random walk with restart (also known as insulated diffusion and personalized PageRank) which better captures the local topology of the interaction network compared with a general heat diffusion process. The process is captured by the steady-state solution as follows:


(1) $$P_{SS}=\alpha \;(I-(1-\alpha )W{)}^{-1}\;P_{0}$$

where $P_{SS}$ represents the vector of propagated values at a steady state, $P_{0}$ is the initial labeling (genes of interest from molecular studies), W is the normalized version of the adjacency matrix of the underlying network (in this implementation W = AD^−1^ , where A is the unnormalized adjacency matrix and D is the diagonal degree matrix of the network), I is the identity matrix, and α denotes the restart probability (here, α = 0.2), which is the probability of returning to the previously visited node, thus controlling the spread through the network.

To create our base network, we merged ReactomeFI (40), CORUM (41), and HIV-human complexes (11). We then performed two independent propagations: one for human genes associated with HIV and the other for the top 10 E3s in this study. For the former, we extracted all genes that were members of pathway terms containing the word “HIV” in the MSigDB pathway database (c2.cp.v7.4.symbols.gmt) and were also members of the base network (*n* = 282). To seed each propagation, genes of interest were labeled in a binary on or off fashion. After propagation, the two propagated networks were integrated by multiplying across them, gene-wise. Such an operation is used to create a gene list ranked to prioritize genes with high scores from both propagated data sets. To control for nodes with high degree (i.e., many connections), which due to their heightened connectivity are biased to receive higher propagation scores, we conducted a permutation test. Specifically, we simulated random propagations by shuffling the positive scores to random genes, repeating this 20,000 times per propagation run. Next, we derived an empirical *P* value by calculating the fraction of random propagation runs greater than or equal to the true propagation run for each gene.

The significant subnetwork was created by extracting genes with a *P*-value ≤0.05 from the base network, requiring they form a singular connected component (i.e., possessed at least one connection to another gene within this set; *n* = 447). The resulting significant subnetwork was clustered into smaller subnetwork clusters using the cluster_walktrap function (iGraph; steps = 10) in conjunction with the cophenetic function in R (stats package) to calculate a distance matrix (82). The cluster_walktrap function was weighed using weights derived from similarity of gene co-membership within Gene Ontology (GO) and pathway (c2.cp.v7.4.symbols.gmt and c5.all.v7.1.symbols.gmt from MSigDB as well as CORUM) terms. The final dendrogram was cut (i.e., clustered) using the dynamicTreeCut package in R (minClusterSize = 7, deepSplit = 4, and method = ‘hybrid’). This resulted in 30 subnetwork clusters (#0–29; Fig. S2). Lastly, GO enrichment analysis (biological process) was performed for each of the 30 resulting subnetwork clusters to identify biological processes associated with each cluster (Fig. S4).

### AZD5582 and GS-9620 treatment and collection of edited activated primary CD4+ T cells to probe for NF-κB activation by western blot

Primary activated CD4+ T cells were isolated, activated, and edited as described above with guides NTC-02, MARCH5-01, TRAF2-01, TRAF3-03, UHRF1-01, and ZFP91-02 (designed by Horizon; Table S2). Eight days after editing, activated primary CD4+ T cells were treated with 100 nM of AZD5582 (a generous gift from Sumit Chanda’s lab) and 100 nM GS-9620 (Cayman Chemical) or untreated with complete media [RPMI-1640 media (Corning) with 10% FBS (Gibco), 50 μg/mL penicillin-streptomycin (Corning), 5 mM sodium pyruvate (Corning), 5 mM 4-(2-hydroxyethyl)-1-piperazineethanesulfonic acid (HEPES) (HyClone), and 20 IU/mL IL-2 (Miltenyi Biotec)]. Cells were collected 24 hours after treatment by resuspension in 2.5× Laemmli Sample Buffer (25 mM Tris pH 6.8, 8% glycerol, 0.8% SDS, 2% 2-mercaptoethanol, and 0.02% bromophenol blue) and incubation for 20 min at 95°C. Ten microliters of the sample was separated by SDS-PAGE on precast Criterion 26-well 4%–20% TGX gels (BioRad) at 90 V for 100 min. Proteins were transferred to PVDF membranes at 4°C for 90 min at 25 milliAmps in 20% methanol transfer buffer. PVDF membranes were blocked with 5% milk in TBST for 1 hour. Blocked membranes were incubated with primary antibodies against p100/p52 (1:1,000) (Cell Signaling Technology), IκBα (1:1,000) (Cell Signaling Technology), or GAPDH (1:1000) (Sigma) at 4°C rocking overnight, washed six times for 5 min each with TBST, and incubated with secondary antibody (goat anti-rabbit IgG-HRP [1:5,000–1:10,000] [Cell Signaling Technology] or goat anti-mouse IgG-HRP [1:5,000] [Cell Signaling Technology]) for 1 hour. Membranes were washed with TBST six times for 5 min and incubated with 2 mL of Pierce ECL western blotting substrate (for anti-IκBα and anti-GAPDH) (Thermo Fisher Scientific) or SuperSignal West Pico PLUS chemiluminescent substrate (for anti-p100/p52) (Thermo Fisher Scientific) for 3–5 min. Blots were exposed with an autoradiography film (Thomas Scientific) and developed with a medical film processor (Konica Minolta Medical & Graphic).

### CRISPR-Cas9 editing and LRA treatment of 11.1, A2, and A72 JLat cells

Editing was performed as described above in activated primary CD4+ T cells with the following exceptions: crRNA guides used were NTC-02, MARCH5-01, TRAF2-01, TRAF3-03, UHRF1-01, and ZFP91-02 (designed by Horizon, Table S2), no stimulation beads were used, and complete media were RPMI-1640 media (Corning) with 10% FBS (Gibco), 50 μg/mL penicillin-streptomycin (Fisher), and 2 mM L-Glutamine (Corning). Then, 300,000 JLat cells were resuspended in 20 µL Lonza electroporation buffer SE (Lonza) and electroporated with code CM-137. Cells were fed with complete media every 2–3 days until drug treatment. Eight days after electroporation with crRNPs, JLat cells were treated with 10 ng/mL TNFalpha (PeproTech), 100 nM AZD5582 (a generous gift from Sumit Chanda’s lab), 100 nM GS-9620 (Cayman Chemical), 10 µg/mL PHA (a generous gift from Melanie Ott’s lab), 16 mM PMA (Ott lab) + 0.5 µM Ionomycin (Ott lab), and 625 nM JQ1 (Ott lab) or untreated with complete media. Cells were collected 24 hours after treatment by resuspension in 1% paraformaldehyde in PBS.

### Flow cytometry and computational analysis of GFP-positive edited 11.1, A2, and A72 JLat cells

Fixed cells were analyzed by an Attune NxT Acoustic Focusing Cytometer (Thermo Fisher), recording all events in a 100-µL sample volume after one 150-µL mixing cycle. Data were exported as FCS3.0 files and analyzed with a consistent template in FlowJo. Cells were gated on live lymphocytes, singlets, and then, the percentage of GFP+ cells was quantified. The data were exported to CSV, including the count and percentage of lymphocytes and the count and percentage of GFP+ cells. Nine replicates (three biological replicates with three technical replicates each) were averaged for the percentage of lymphocytes and percentage of GFP+ cells, and standard deviation was calculated. Data were removed if the average percentage of lymphocytes was below the viability cutoff of 30%. For the seven guides/two treatments experiment (Fig. 4B, Fig. S5B), fold change and *P* value were calculated by comparison with the NTC of the same treatment within the same cell line. Significance was defined as a fold change ≥ 1.5 and a *P* value <0.05. For the three guides/seven treatments experiment (Fig. 4C; Fig. S5C and D), fold change and *P* value were calculated by comparison with the untreated condition of the same gene knockout within the same cell line. Significance was defined as a fold change ≥ 1.5 and a *P* value <0.05.

### CRISPR-Cas9 RNP generation for electroporation in resting primary CD4+ T cells

Lyophilized tracrRNA (Horizon) and crRNA were resuspended at 160 µM in 10 mM Tris-HCl (7.4 pH) with 150 mM KCl. Pre-formed crRNPs were made by incubating 1.5 µL of 160 µM tracrRNA with 1.5 µL of 160 µM guide RNA and incubating for 30 min at 37°C. Next, 1.2 µL of 100 nmol/µL single-stranded donor oligonucleotides (ssODN; sequence: TTAGCTCTGTTTACGTCCCAGCGGGCATGAGAGTAACAAGAGGGTGTGGTAATATTACGGTACCGAGCACTATCGATACAATATGTGTCATACGGACACG, IDT) was added and incubated at 37°C for 5 min. Next, 3 µL of 40 µM Cas9 (Macrolab) was added slowly taking care to avoid precipitation, pipetting up and down several times to ensure complete resuspension of the RNP complex, and incubated for 15 min at 37°C. crRNPs were aliquoted at 7.2 µL and frozen at −80°C for up to 6 months before use as described in the next section. Along with guides targeting genes of interest (TRAF2-01 and UHRF1-01; Table S2), each experiment also included a non-targeting control guide (Horizon NTC#3, U-007503-01; Table S2) and a mixture of two guides, targeting the HIV-1 GAG region (HIV-GAG-01 and HIV-GAG-02; Table S2).

### Modeling effect of gene deletions on HIV latency reversal in resting primary CD4+ T cells

Primary human CD4+ T cells were isolated from PBMC-enriched leukapheresis products (Leukopaks) from six healthy donors, following informed written consent (StemCell Technologies), by magnetic negative selection (StemCell, 17952) to avoid activation. These cells were cultured in resting-cell complete RPMI media [rcRPMI; RPMI-1640 media (Corning) with 10% FBS (Gibco), 50 μg/mL penicillin-streptomycin (Corning), 5 mM sodium pyruvate (Corning), 5 mM 4-(2-hydroxyethyl)−1-piperazineethanesulfonic acid (HEPES) (HyClone), 10 IU/mL IL-2 (Miltenyi Biotec), and 5 ng/mL IL-7 (R&D Systems)]. The next day, before performing HIV infection, the expression levels of cellular activation markers CD25 and CD69 were measured on unstimulated CD4+ T cells by flow cytometry and compared with control CD4+ T cells activated with anti-CD3/anti-CD2/anti-CD28 beads (T Cell Activation and Stimulation Kit, Miltenyi) at a 1:1 bead:cell ratio (Fig. S6A). Unstimulated primary CD4+ T cells were spinoculated with a replication-competent HIV strain (LAI.2, subtype B, CXCR4 tropic) as described above. The infected cells were immediately cultured at 5e6 cells/mL density in rcRPMI media in the presence of 10 µM Saquinavir for 5 days to promote viral integration and latency as described in the Greene model of latency (55). The media were replenished after every 2 days. After 5 days of culturing in Saquinavir, the cells were electroporated with crRNPs by mixing 7.2 µL of crRNP-ssODN solution with 1.5e6 unstimulated HIV-infected CD4+ T cells in 18 µL of Lonza electroporation buffer P2. The electroporation was done in Lonza 96-well nucleocuvette plates with code EH-110 on a Lonza 4D nucleofector core unit and Lonza 96-well shuttle device (Lonza). After the nuke, the cells were again cultured in the presence of saquinavir for 5 more days to allow degradation of the proteins encoded by targeted genes. After which, the cells were washed and moved to crRPMI media containing 60 µM Raltegravir, a potent HIV integrase inhibitor to allow reactivation of the latent virus while preventing spreading infection. After 2 days of culture in the presence of Raltegravir, approximately 10,000 cells were collected from each sample for TIDE analysis and the remaining cells were pelleted down for intracellular P24 staining. The supernatant was collected, frozen, and stored at −80°C until use for p24 ELISA.

### Measurement of latency reversal in edited resting primary CD4+ T cells by intracellular p24 staining and flow cytometry

Pelleted cells were first stained extracellularly with Ghost Dye Violet 510 (Tonbo Biosciences) for live-dead discrimination. Briefly, cells were pelleted at 300 × *g* for 5 min in a v-bottom 96-well plate and the media were removed. Cells were suspended in a 1:500 dilution of the antibody in MACS buffer (0.5% bovine serum albumin and 2 mM EDTA in PBS) at a concentration of roughly 10e3 cells/µL and incubated for 20 min at RT. Cells were pelleted again, washed with MACS buffer, and suspended in 1% paraformaldehyde-PBS for fixation prior to intracellular staining. Intracellular staining was performed on infected, fixed resting primary CD4+ T cell populations with 1:100 dilution of p24-FITC antibody (KC57, Beckman Coulter) using a FOXP3 Fix/Perm buffer set (BioLegend) as per the manufacturer’s instructions. After intracellular p24 staining, the cells were resuspended in 1% paraformaldehyde PBS for flow cytometric analysis on an Attune NxT Acoustic Focusing Cytometer (Thermo Fisher), recording all events in a 180-µL sample volume after one 200-µL mixing cycle. Data were exported as FCS3.0 files and analyzed in FlowJo. Cells were gated on lymphocytes, side-scatter singlets, forward-scatter singlets, live cells, and then, the percentage of p24+ cells was quantified (Fig. S6B). The data were exported to CSV, including the count and percentage of live cells and the count and percentage of p24+ cells. Three technical replicates for each of the six biological replicates were averaged, and standard deviation was calculated. The fold change and *P* value were calculated by comparison with the NTC of the same donor (Fig. 5C).

### Measurement of the effects of gene deletions on virus production from latently infected resting CD4+ T cells by supernatant p24 ELISA

Latency reversal and HIV-1 production were measured by performing p24-ELISA with the culture supernatants using the Lenti-X p24 Rapid Titer Kit (TaKaRa). Briefly, the culture supernatant from the edited and control samples was diluted 1:40-fold in the complete DMEM medium used for ELISA as per the manufacturer’s instructions. The OD_450_ value for the blank control was subtracted from the value of each sample. Three technical replicates for each of the six biological replicates were averaged, and standard deviation was calculated. The fold change in supernatant p24 (proxy for HIV production) and *P* value were calculated by comparison with the NTC of the same donor.

### TIDE analysis of edited primary CD4+ T cells and JLat cells

The efficiency of the gene knockout in each case was assessed by performing TIDE analysis with the cell lysates (26). Briefly, 10 µL of cell suspension was added to 20 µL of QuickExtract buffer (Lucigen QE09050) in a PCR plate and heated at 65°C for 6 min and 98°C for 2 min to prepare cell lysates containing genomic DNA. PCR amplification over the cut site was performed using this genomic DNA to generate a template for sanger sequencing, which was then analyzed for indel percentage using the TIDE webtool. Primers were designed with the Primer3 tool in Benchling. PCR amplification was performed using NEBNext Ultra II Q5 2X Master Mix (New England Biolabs M0544L), 10 µM primer pair, and approximately 100 ng template DNA. PCR amplicons were subsequently sent for cleanup and Sanger sequencing. Mutational efficiency was determined by comparison of non-targeting and gene-targeting sample chromatograms using the TIDE Web Tool (59). The TIDE output calculates the percentage of insertions and deletions (indels) from these chromatograms. The total efficiency of indel generation provides a reasonable estimate of knockout percentage in a cell population following crRNP treatment.

## Data Availability

Mass spectrometry data files (raw and search results) have been deposited to the ProteomeXchange Consortium (http://proteomecentral.proteomexchange.org) via the PRIDE partner repository with data set identifier PXD028127 (83). This article analyzes existing publicly available data, and we have provided the citations to the original publications from where we downloaded this data. All data reported in this paper will be shared by the corresponding author upon request. Any additional information required to reanalyze the data reported in this paper is available from the corresponding author upon request.

## SUPPLEMENTAL MATERIAL

Supplemental material mbio.02222-23-s0001.pdf Supplemental table captions, supplemental figures, and extended data.

Table S1 mbio.02222-23-s0002.xlsx Proteins identified by LC-MS/MS analysis of primary activated CD4+ T cells.

Table S2 mbio.02222-23-s0003.xlsx CRISPR guides used for gene knockout by electroporation with CRISPR-Cas9 ribonucleoproteins (crRNPs).

Table S3 mbio.02222-23-s0004.xlsx Raw and analyzed data from CRISPR-Cas9 knockout spreading HIV-1 infection assay.

Table S4 mbio.02222-23-s0005.xlsx Network propagation of HIV pathways and E3 ubiquitin ligases that functionally affected HIV infection in our CRISPR-Cas9 knockout spreading HIV-1 infection assay.

Table S5 mbio.02222-23-s0006.xlsx Raw and analyzed data from CRISPR-Cas9 knockouts in JLat models of HIV latency.

Table S6 mbio.02222-23-s0007.xlsx Raw and analyzed data from CRISPR-Cas9 knockouts in a resting primary human CD4+ T cell model of HIV latency.

ASM does not own the copyrights to Supplemental Material that may be linked to, or accessed through, an article. The authors have granted ASM a non-exclusive, world-wide license to publish the Supplemental Material files. Please contact the corresponding author directly for reuse.
