# Supplementary material for: CRISPR-Cas9 screen of E3 ubiquitin ligases identifies TRAF2 and UHRF1 as regulators of HIV latency in primary human T cells
Source: mBio. 2024 Feb 27;15(4):e02222-23. doi: 10.1128/mbio.02222-23 (PMC11005436; doi:10.1128/mbio.02222-23)
Supplement: Supplemental material — Supplemental table captions, supplemental figures, and extended data. [file mbio.02222-23-s0001.pdf]

CRISPR-Cas9 screen of E3 ubiquitin ligases identifies TRAF2 and UHRF1 as regulators of HIV latency in primary human T cells

Ujjwal Rathore,[1,2,3] Paige Haas,[1,4,5] Vigneshwari Easwar Kumar,[1,2,3] Joseph Hiatt,[1,2,3,6,7] Kelsey M. Haas,[1,4,5] Mehdi Bouhaddou,[1,4,5] Danielle L. Swaney,[1,4,5] Erica Stevenson,[1,4,5] Lorena Zuliani-Alvarez,[1,4,5] Michael J. McGregor,[1,4,5] Autumn Turner-Groth,[1] Charles Ochieng' Olwal,[8,9] Yaw Bediako,[8,9] Hannes Braberg,[1,4,5] Margaret Soucheray,[1,4,5] Melanie Ott,[1] Manon Eckhardt,[1,4,5] Judd F. Hultquist,[10,11] Alexander Marson,[1,2,3,12,13,14,15,16] Robyn M. Kaake,[1,4,5] Nevan J. Krogan[1,4,5]

Affiliations:

[1]Gladstone Institutes, San Francisco, California, USA

[2]Department of Microbiology and Immunology, University of California, San Francisco, California, USA

[3]Innovative Genomics Institute, University of California, Berkeley, California, USA

[4]Quantitative Biosciences Institute (QBI), University of California, San Francisco, California, USA

[5]Department of Cellular and Molecular Pharmacology, University of California, San Francisco, California, USA

[6]Medical Scientist Training Program, University of California, San Francisco, California, USA

[7]Biomedical Sciences Graduate Program, University of California, San Francisco, California, USA

[8]West African Centre for Cell Biology of Infectious Pathogens (WACCBIP), College of Basic and Applied Sciences, University of Ghana, Accra, Ghana

[9]Department of Biochemistry, Cell & Molecular Biology, College of Basic & Applied Sciences, University of Ghana, Accra, Ghana

[10]Division of Infectious Diseases, Northwestern University Feinberg School of Medicine, Chicago, Illinois, USA

[11]Center for Pathogen Genomics and Microbial Evolution, Institute for Global Health, Northwestern University Feinberg School of Medicine, Chicago, Illinois, USA

[12]Department of Medicine, University of California, San Francisco, California, USA

[13]Diabetes Center, University of California, San Francisco, California, USA

[14]UCSF Helen Diller Family Comprehensive Cancer Center, University of California, San Francisco, California, USA

[15]Parker Institute for Cancer Immunotherapy, University of California, San Francisco, California, USA

[16]Institute for Human Genetics, University of California, San Francisco, California, USA

Author List Footnotes:

Ujjwal Rathore and Paige Haas contributed equally to this article. Author order was determined based on project lead status at the time of final submission.

Corresponding Author(s) for Materials and Correspondence: N.J.K.: nevan.krogan@ucsf.edu; R.M.K.: robyn.kaake@gladstone.ucsf.edu

SUPPLEMENTAL TABLE CAPTIONS

Table S1. Proteins identified by LC-MS/MS analysis of primary activated CD4+ T cells. Separate Excel file.

Table S2. CRISPR guides used for gene knockout by electroporation with CRISPR-Cas9 ribonucleoproteins (crRNPs). Separate Excel file.

Table S3. Raw and analyzed data from CRISPR-Cas9 knockout spreading HIV-1 infection assay. Separate Excel file.

Table S4. Network propagation of HIV pathways and E3 ubiquitin ligases that functionally affected HIV infection in our CRISPR-Cas9 knockout spreading HIV-1 infection assay. Separate Excel file.

Table S5. Raw and analyzed data from CRISPR-Cas9 knockouts in JLat models of HIV latency. Separate Excel file.

Table S6. Raw and analyzed data from CRISPR-Cas9 knockouts in a resting primary human CD4+ T cell model of HIV latency. Separate Excel file.

SUPPLEMENTARY FIGURES

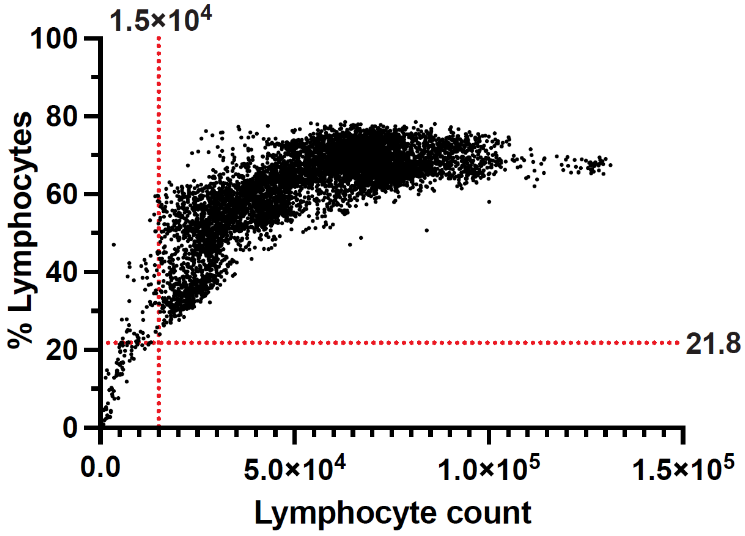

Fig. S1. Scatter plot showing the flow cytometry analysis of the % lymphocytes by the lymphocyte count of all knockout events from all donors, guides, technical replicates, and all three days of cell collection (2, 4, and 6 days after HIV-1 infection). Using this plot to estimate viability, we filtered out the cells that fell below 15000 lymphocyte count and that were less than 21.8% lymphocytes. In total, this filtered out 1% of the total events.

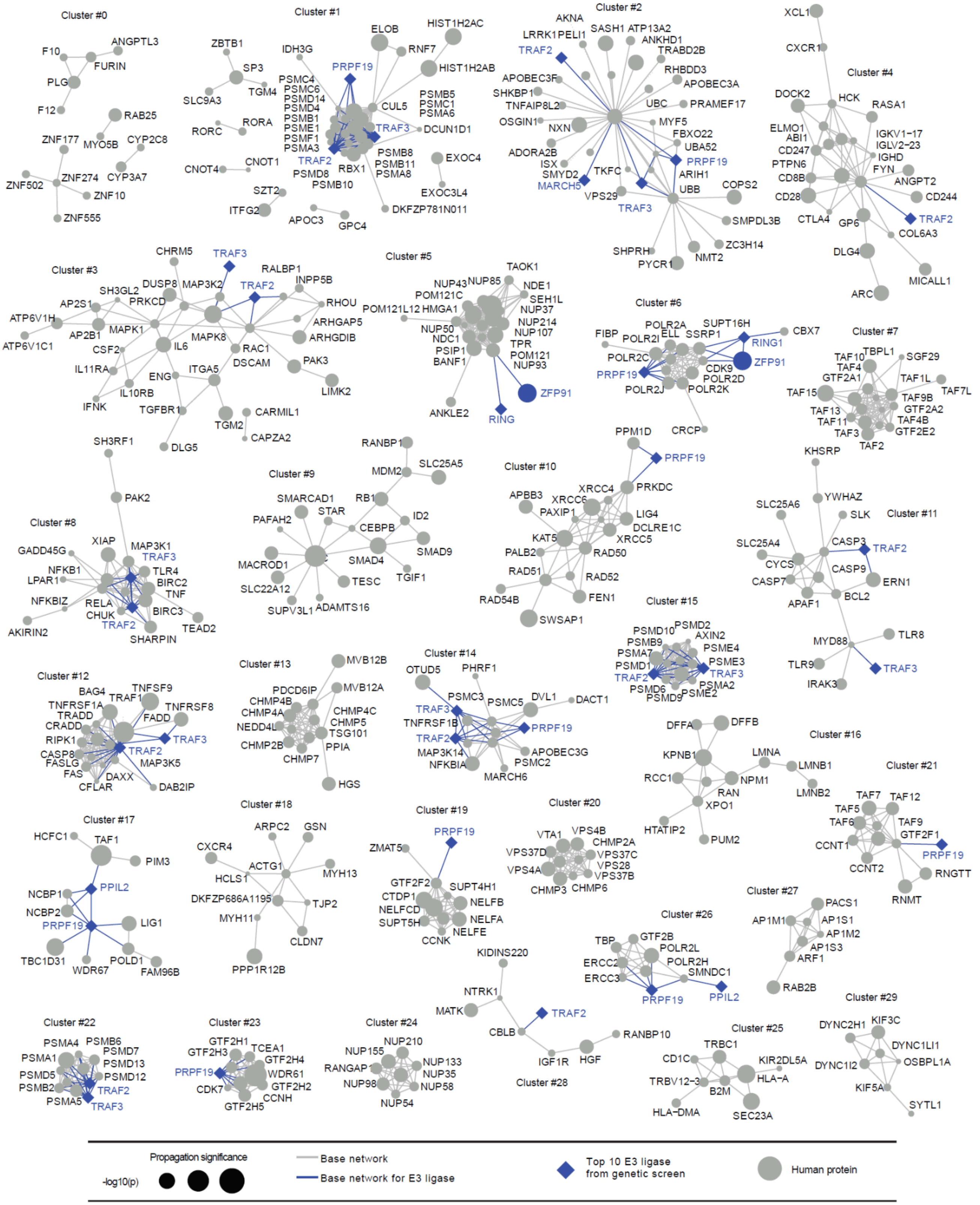

Cluster #0
F10
ANGPTL3
FURIN
PLG
F12
RAB25
ZNF177
MYO5B
CYP2C8
ZNF274
CYP3A7
ZNF502
ZNF10
ZNF555
Cluster #1
ZBTB1
IDH3G
ELOB
HIST1H2AC
RNF7
SP3
PRPF19
HIST1H2AB
SLC9A3
TGM4
PSMC4
PSMC6
PSMD14
PSMD4
PSMB1
PSME1
PSMF1
PSMA3
CUL5
PSMB5
PSMC1
PSMA6
RORC
RORA
TRAF3
DCUN1D1
CNOT4
CNOT1
TRAF2
RBX1
PSMB8
PSMB11
PSMA8
PSMD8
PSMB10
EXOC4
SZT2
EXOC3L4
ITFG2
APOC3
GPC4
DKFZP781N011
Cluster #2
AKNA
SASH1
ATP13A2
LRRK1
PELI1
ANKHD1
TRAF2
TRABD2B
APOBEC3F
RHBDD3
APOBEC3A
SHKBP1
TNFAIP8L2
UBC
PRAMEF17
OSGIN1
NXN
MYF5
FBXO22
ADORA2B
UBA52
ISX
PRPF19
SMYD2
TKFC
ARIH1
MARCH5
UBB
COPS2
VPS29
TRAF3
SMPDL3B
SHPRH
ZC3H14
PYCR1
NMT2
Cluster #4
XCL1
CXCR1
DOCK2
HCK
RASA1
ELMO1
ABI1
IGKV1-17
IGLV2-23
CD247
IGHD
PTPN6
FYN
CD8B
ANGPT2
CD28
CD244
CTLA4
GP6
TRAF2
COL6A3
DLG4
MICALL1
ARC
Cluster #3
CHRM5
TRAF3
RALBP1
SH3GL2
DUSP8
MAP3K2
TRAF2
INPP5B
AP2S1
PRKCD
RHOU
ATP6V1H
MAPK1
ARHGAP5
AP2B1
MAPK8
ARHGDIB
ATP6V1C1
CSF2
IL6
RAC1
DSCAM
IL11RA
ITGA5
PAK3
ENG
IL10RB
LIMK2
IFNK
TGFBR1
CARMIL1
TGM2
CAPZA2
DLG5
Cluster #5
TAOK1
NUP43
NUP85
NDE1
POM121C
SEH1L
POM121L12
HMGA1
NUP37
NUP214
NUP50
NUP107
NDC1
TPR
PSIP1
POM121
BANF1
NUP93
ZFP91
ANKLE2
RING
Cluster #6
POLR2A
SUPT16H
CBX7
FIBP
POLR2I
ELL
SSRP1
RING1
POLR2C
ZFP91
CDK9
PRPF19
POLR2D
POLR2J
POLR2K
CRCP
Cluster #7
TBPL1
TAF10
SGF29
TAF4
GTF2A1
TAF1L
TAF7L
TAF15
TAF9B
TAF13
GTF2A2
TAF11
TAF4B
TAF3
GTF2E2
TAF2
SH3RF1
PAK2
Cluster #8
XIAP
MAP3K1
GADD45G
TRAF3
NFKB1
TLR4
LPAR1
BIRC2
TNF
NFKBIZ
RELA
BIRC3
CHUK
AKIRIN2
TRAF2
TEAD2
SHARPIN
Cluster #9
RANBP1
SLC25A5
MDM2
SMARCAD1
RB1
STAR
ID2
PAFAH2
CEBPB
SMAD9
SMAD4
MACROD1
TGIF1
TESC
SLC22A12
SUPV3L1
ADAMTS16
PPM1D
CRCP
PRPF19
Cluster #10
PRKDC
XRCC4
APBB3
XRCC6
PAXIP1
LIG4
DCLRE1C
KAT5
XRCC5
PALB2
RAD50
RAD51
RAD52
RAD54B
FEN1
SWSAP1
KHSRP
YWHAZ
SLC25A6
SLK
Cluster #11
SLC25A4
CASP3
TRAF2
CYCS
CASP9
CASP7
ERN1
APAF1
BCL2
Cluster #15
PSMD10
PSMD2
AXIN2
PSMB9
PSME4
PSMA7
PSME3
PSMD1
TRAF3
TRAF2
PSMA2
PSMD6
PSME2
PSMD9
MYD88
TLR8
TLR9
TRAF3
IRAK3
Cluster #12
BAG4
TNFSF9
TRAF1
TNFRSF1A
TNFRSF8
TRADD
FADD
CRADD
TRAF3
RIPK1
CASP8
TRAF2
FASLG
MAP3K5
FAS
DAXX
CFLAR
DAB2IP
Cluster #13
MVB12B
MVB12A
PDCD6IP
CHMP4B
CHMP4A
CHMP4C
CHMP5
NEDD4L
TSG101
CHMP2B
PPIA
CHMP7
HGS
Cluster #14
OTUD5
PHRF1
PSMC3
PSMC5
DVL1
DACT1
TRAF3
TNFRSF1B
PRPF19
TRAF2
APOBEC3G
MAP3K14
PSMC2
NFKBIA
MARCH6
DFFA
DFFB
Cluster #16
KPNB1
LMNA
LMNB1
RCC1
NPM1
LMNB2
RAN
XPO1
HTATIP2
PUM2
Cluster #21
TAF7
TAF12
TAF5
TAF6
TAF9
GTF2F1
CCNT1
PRPF19
CCNT2
RNGTT
RNMT
Cluster #17
HCFC1
TAF1
PIM3
PPIL2
NCBP1
NCBP2
LIG1
PRPF19
TBC1D31
POLD1
WDR67
FAM96B
Cluster #18
ARPC2
GSN
CXCR4
ACTG1
HCLS1
MYH13
DKFZP686A1195
TJP2
MYH11
CLDN7
PPP1R12B
Cluster #19
PRPF19
ZMAT5
GTF2F2
SUPT4H1
CTDP1
NELFB
NELFCD
NELFA
SUPT5H
NELFE
CCNK
Cluster #20
VTA1
VPS4B
CHMP2A
VPS37D
VPS37C
VPS4A
VPS28
VPS37B
CHMP3
CHMP6
Cluster #27
PACS1
AP1M1
AP1S1
AP1M2
AP1S3
ARF1
RAB2B
Cluster #26
TBP
GTF2B
POLR2L
ERCC2
POLR2H
ERCC3
SMNDC1
PRPF19
PPIL2
KIDINS220
NTRK1
MATK
TRAF2
CBLB
RANBP10
IGF1R
HGF
Cluster #28
Cluster #29
DYNC2H1
KIF3C
DYNC1LI1
DYNC1I2
OSBPL1A
KIF5A
SYTL1
Cluster #25
TRBC1
CD1C
KIR2DL5A
HLA-A
TRBV12-3
B2M
HLA-DMA
SEC23A
Cluster #22
PSMA4
PSMB6
PSMD7
PSMA1
PSMD13
PSMD5
PSMD12
PSMB2
TRAF2
PSMA5
TRAF3
Cluster #23
GTF2H1
TCEA1
GTF2H3
GTF2H4
PRPF19
WDR61
GTF2H2
CDK7
CCNH
GTF2H5
Cluster #24
NUP210
NUP155
NUP133
RANGAP1
NUP35
NUP98
NUP58
NUP54
Propagation significance
-log10(p)
Base network
Base network for E3 ligase
Top 10 E3 ligase from genetic screen
Human protein

Fig. S2. Final clustered subnetworks from network propagation analysis. Integrative network propagation analysis was performed by merging networks propagated with either genes associated with HIV pathogenesis or the top E3s identified in this study (see Methods). The significant subnetwork was created by extracting genes (grey circles) with p-value <= 0.05 from the base network, requiring they form a singular connected component (i.e. possessed at least one connection to another gene within this set; n=447). The circle size corresponds to the -log10(p-value) from the propagation analysis. The resulting significant subnetwork was clustered into 30 smaller subnetwork clusters and E3s with known connections to each cluster were overlaid onto each cluster (blue diamonds and lines).

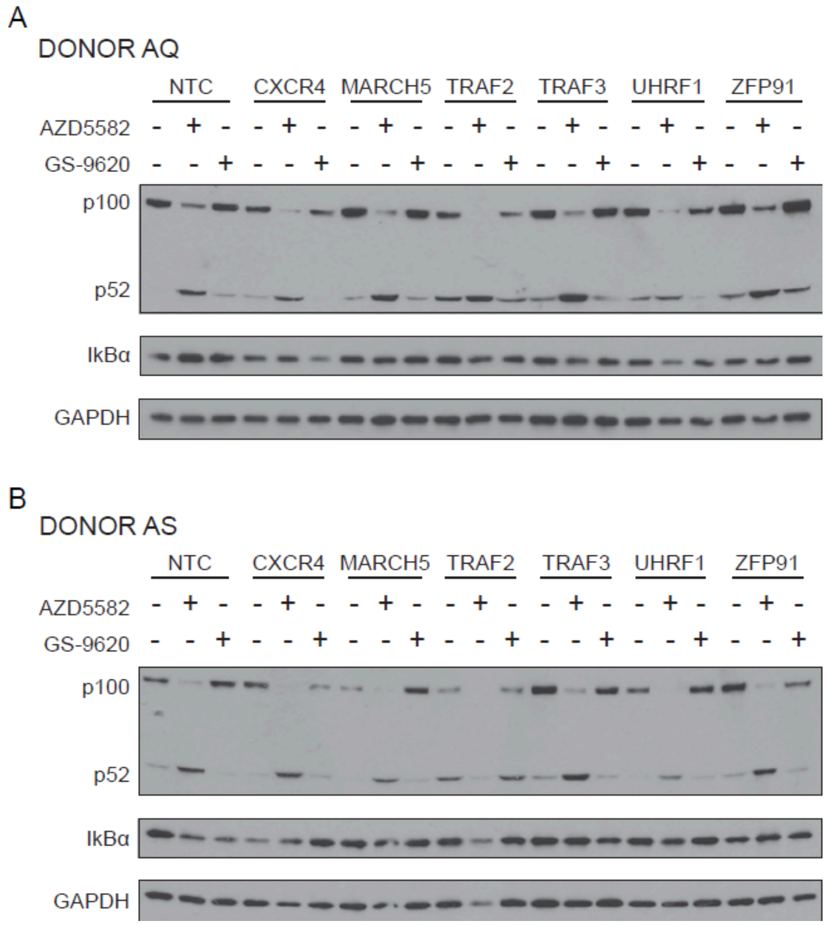

Fig. S3. Western blot analysis of non-canonical and canonical NF-κB activation in the remaining two of three donors. a. Western blot analysis probing for non-canonical NF-κB activation (marked by the processing of p100 to p52), and non-canonical NF-κB activation (marked by IκBα degradation), with a GAPDH loading control in gene knockouts in primary activated CD4+ T cells from healthy human donor AQ. **b**. Same as in (A) but from healthy human donor AS.

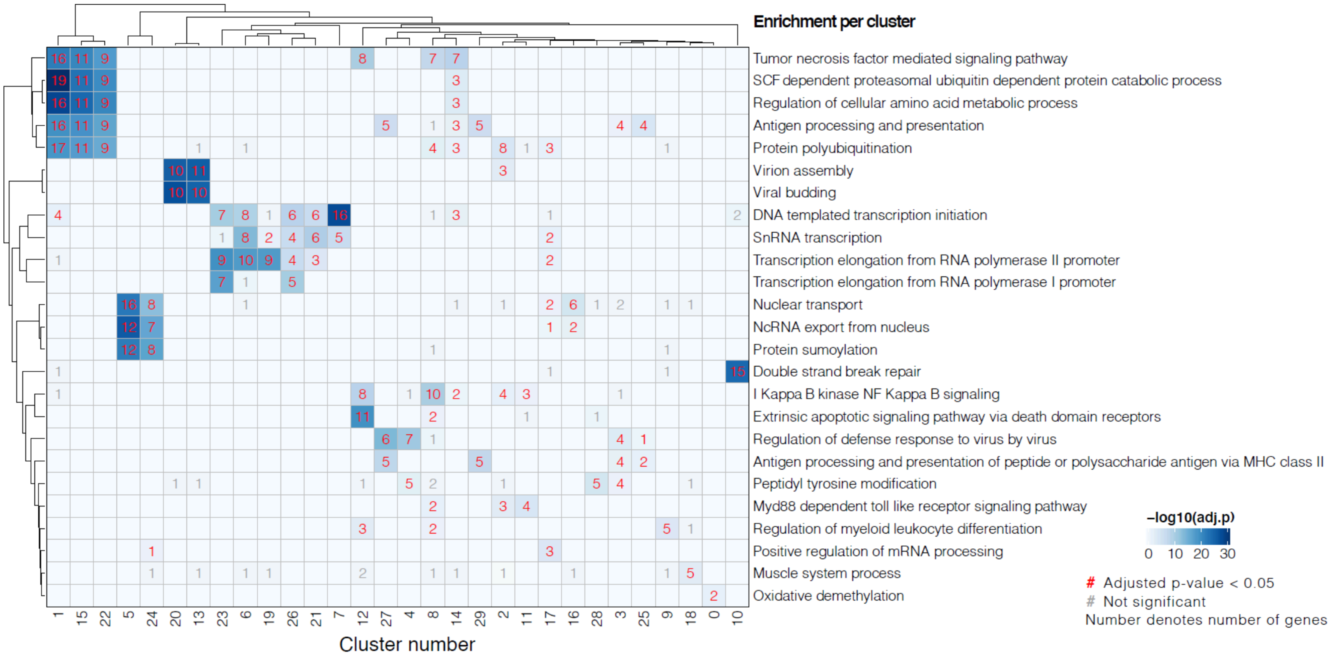

Fig. S4. Enrichment for subnetwork clusters (annotated along with the columns) identified in Supplemental Fig. 3. Gene Ontology (GO) enrichment analysis (biological process) was performed for each of the 30 resulting subnetwork clusters to identify biological processes associated with each cluster. Color corresponds to the -log10(adjusted p-values) from the GO Biological Process enrichment analysis. Numbers denote the number of genes enriched in each cluster for each term; if significant (adjusted p-value < 0.05) they are colored red, otherwise grey.

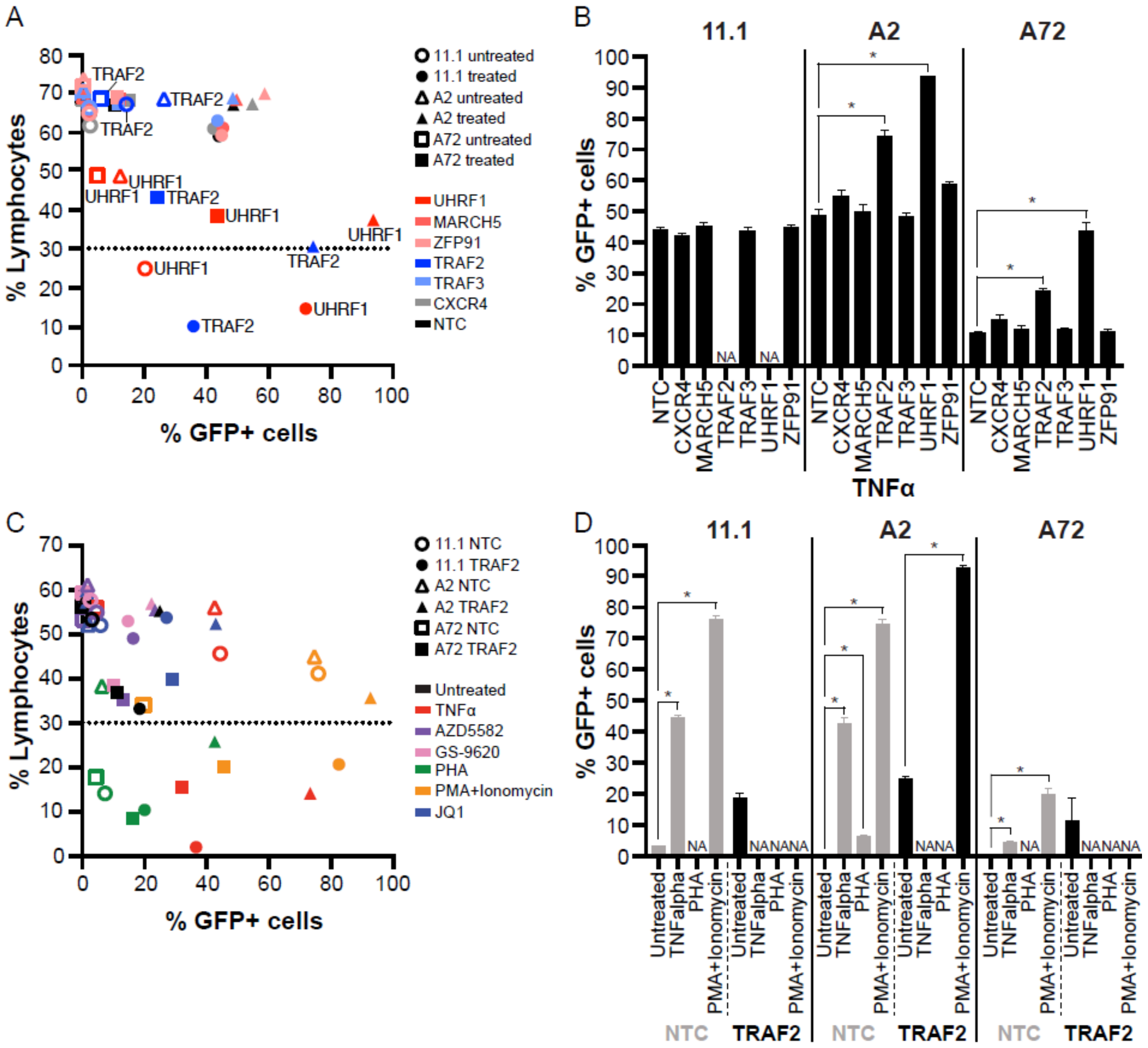

Fig. S5. Gene knockouts and LRA treatments in JLat models. a. Flow cytometry quantification of viability (% lymphocytes) relative to HIV latency reversal (%GFP+ cells) for gene knockouts treated with TNFα or untreated in JLat cell lines 11.1, A2, and A72. A viability cutoff was set at 30% lymphocytes. **b**. Flow cytometry quantification of latency reversal (%GFP+ cells) for gene knockouts treated with TNFα in JLat cell lines 11.1, A2, and A72. Samples that did not pass the viability cutoff, and were removed and denoted "NA". Significance was defined as a fold change ≥1.5 and a p-value <0.05 compared to the TNFα-treated NTC within the same cell line. **c**. Flow cytometry quantification of

viability (% lymphocytes) relative to HIV latency reversal (% GFP+ cells) for NTC or TRAF2 knockouts treated with a panel of latency-reversing agents (LRAs) in JLat cell lines 11.1, A2, and A72. A viability cutoff was set at 30% lymphocytes. **d**. Flow cytometry quantification of latency reversal (% GFP+ cells) for NTCs and TRAF2 knockouts that were untreated or treated with the LRAs TNFalpha, PHA, or PMA+Ionomycin in JLat cell lines 11.1, A2, and A72. Samples that did not pass the viability cutoff, and were removed and denoted “NA”. Significance was defined as a fold change ≥1.5 and a p-value <0.05 compared to the untreated condition of the same gene knockout within the same cell line.

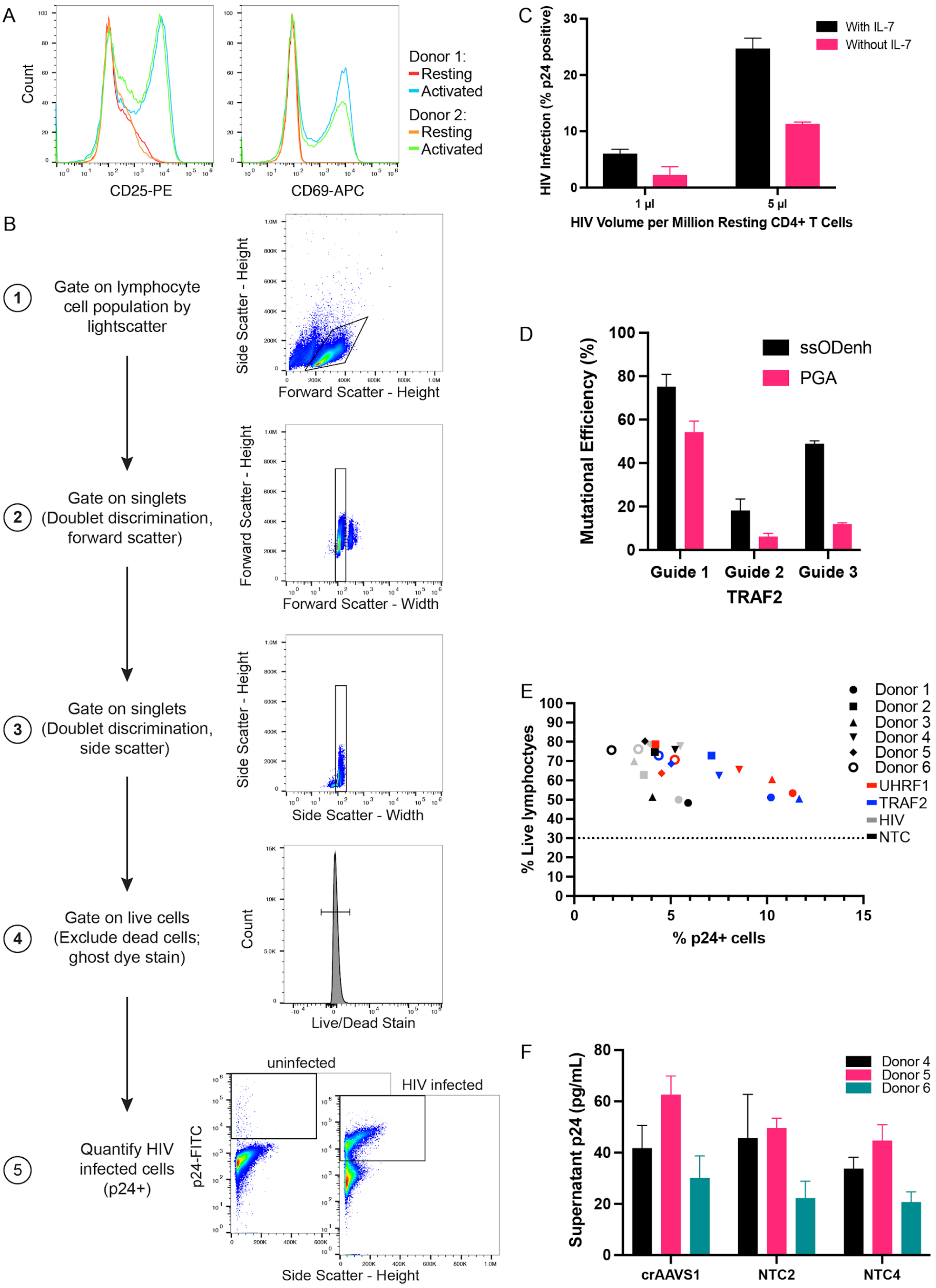
A
Count
CD25-PE
CD69-APC
Donor 1:
Resting
Activated
Donor 2:
Resting
Activated
B
1 Gate on lymphocyte cell population by lightscatter
2 Gate on singlets (Doublet discrimination, forward scatter)
3 Gate on singlets (Doublet discrimination, side scatter)
4 Gate on live cells (Exclude dead cells; ghost dye stain)
5 Quantify HIV infected cells (p24+)
Side Scatter - Height
Forward Scatter - Height
Forward Scatter - Width
Side Scatter - Width
Count
Live/Dead Stain
uninfected
HIV infected
p24-FITC
C
HIV Infection (% p24 positive)
With IL-7
Without IL-7
1 µl
5 µl
HIV Volume per Million Resting CD4+ T Cells
D
Mutational Efficiency (%)
ssODenh
PGA
Guide 1
Guide 2
Guide 3
TRAF2
E
% Live lymphoctyes
% p24+ cells
Donor 1
Donor 2
Donor 3
Donor 4
Donor 5
Donor 6
UHRF1
TRAF2
HIV
NTC
F
Supernatant p24 (pg/mL)
Donor 4
Donor 5
Donor 6
crAAVS1
NTC2
NTC4

Fig. S6. Gene knockouts in a primary resting CD4+ T cell latency model. a. Flow cytometry quantification of CD25 and CD69 activation markers' expression on primary human CD4+ T cells with and without activation. As expected, CD25 and CD69 are expressed only on activated T cells. **b.** Gating strategy for flow cytometry analysis. In step 5, representative HIV-infected (right) and uninfected control (left) are shown. A consistent template was used throughout analysis. Briefly, cells were gated on lymphocytes, side-scatter singlets, forward-scatter singlets, live cells, and then the percentage of p24+ cells was quantified. **c**. Flow cytometry quantification of HIV infection (% p24+ cells) in the presence or absence of IL-7. **d**. Quantification of TRAF2 knockout by sequencing in presence of two different electroporation enhancers. Sanger sequencing was analyzed for mutational efficiency by TIDE. **e**. Flow cytometry quantification of viability (% live lymphocytes) relative to HIV latency reversal (%p24+ cells) for gene knockouts in primary resting CD4+ T cells from six healthy human donors. A viability cutoff was set at 30% live lymphocytes. **f**. ELISA quantification of HIV production (supernatant p24 (pg/ml)) by primary resting CD4+ T cells from three healthy human donors treated with additional control crRNPs.

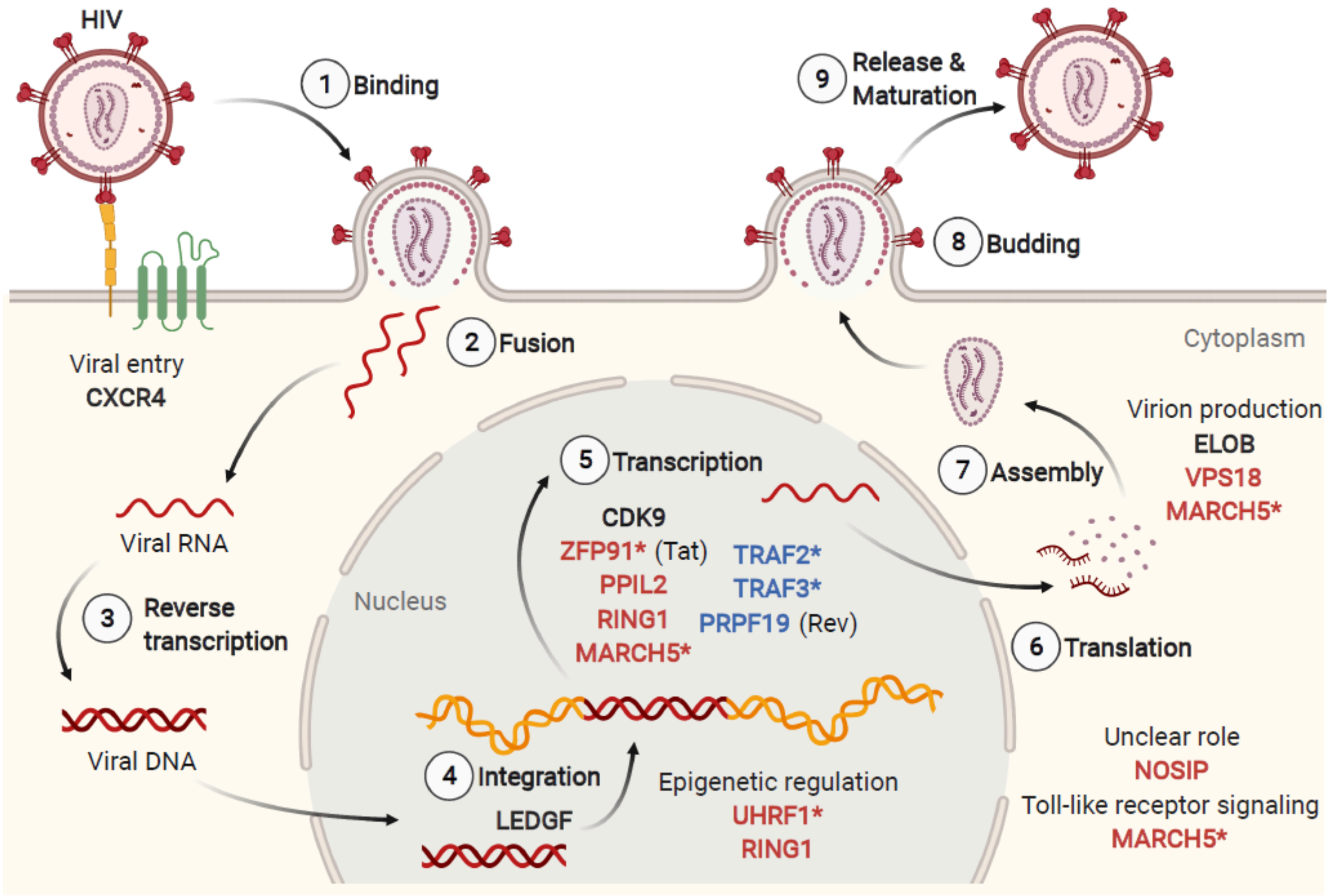

Fig. S7. Potential roles for 10 E3 hits in HIV infection.

EXTENDED DATA

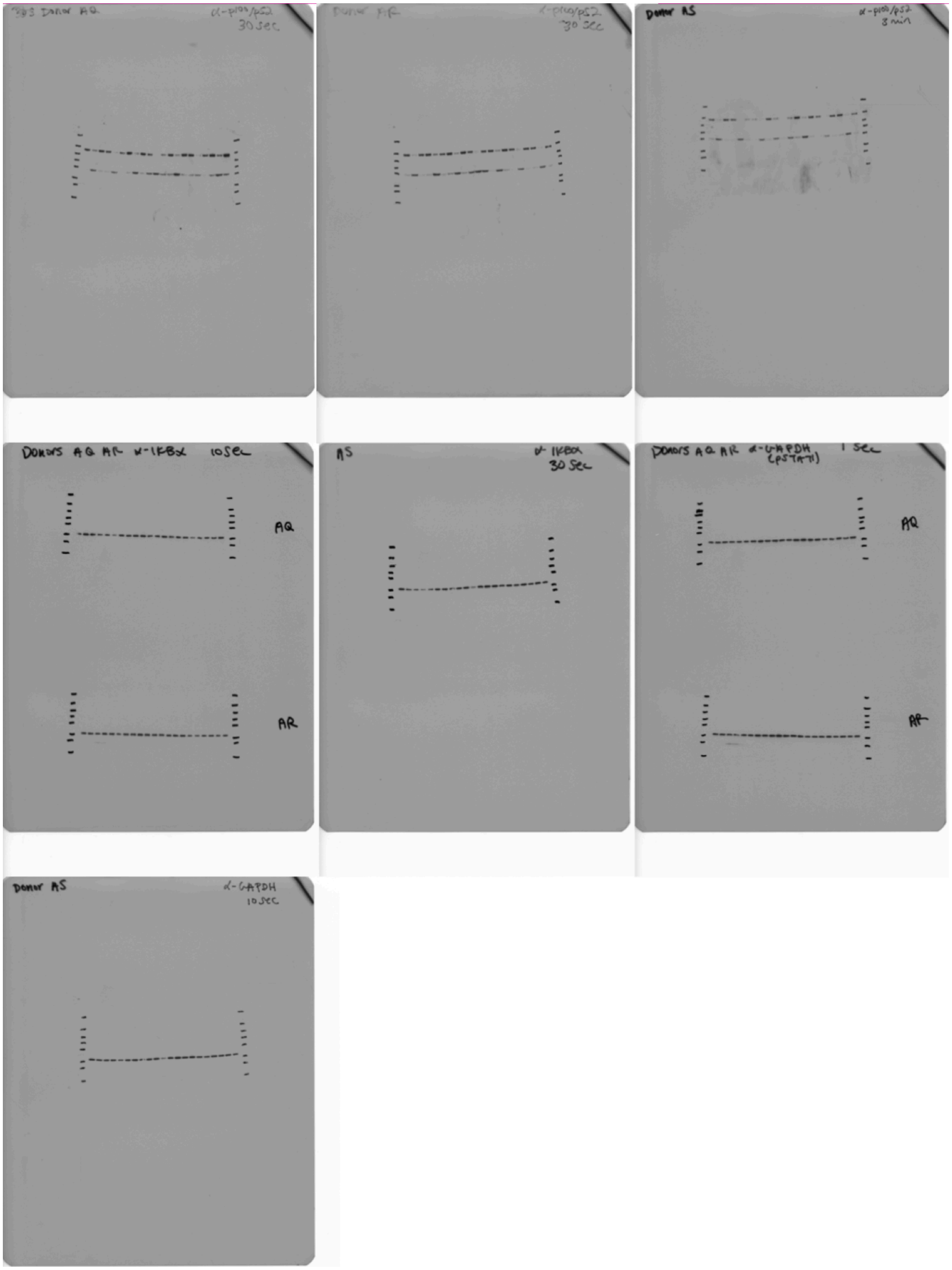

Extended Data. Raw Western blot images, related to Fig. 3 and Fig.S3.
